# Effects of Prism Eyeglasses on Objective and Subjective Fixation Disparity

**DOI:** 10.1371/journal.pone.0138871

**Published:** 2015-10-02

**Authors:** Volkhard Schroth, Roland Joos, Wolfgang Jaschinski

**Affiliations:** 1 University of Applied Sciences and Arts Northwestern Switzerland, Olten, Switzerland; 2 Leibniz Research Centre of Working Environment and Human Factors, Dortmund, Germany; UMR8194, FRANCE

## Abstract

In optometry of binocular vision, the question may arise whether prisms should be included in eyeglasses to compensate an oculomotor and/or sensory imbalance between the two eyes. The corresponding measures of objective and subjective fixation disparity may be reduced by the prisms, or the adaptability of the binocular vergence system may diminish effects of the prisms over time. This study investigates effects of wearing prisms constantly for about 5 weeks in daily life. Two groups of 12 participants received eyeglasses with prisms having either a base-in direction or a base-out direction with an amount up to 8 prism diopters. Prisms were prescribed based on clinical fixation disparity test plates at 6 m. Two dependent variables were used: (1) subjective fixation disparity was indicated by a perceived offset of dichoptic nonius lines that were superimposed on the fusion stimuli and (2) objective fixation disparity was measured with a video based eye tracker relative to monocular calibration. Stimuli were presented at 6 m and included either central or more peripheral fusion stimuli. Repeated measurements were made without the prisms and with the prisms after about 5 weeks of wearing these prisms. Objective and subjective fixation disparity were correlated, but the type of fusion stimulus and the direction of the required prism may play a role. The prisms did not reduce the fixation disparity to zero, but induced significant changes in fixation disparity with large effect sizes. Participants receiving base-out prisms showed hypothesized effects, which were concurrent in both types of fixation disparity. In participants receiving base-in prisms, the individual effects of subjective and objective effects were negatively correlated: the larger the subjective (sensory) effect, the smaller the objective (motor) effect. This response pattern was related to the vergence adaptability, i.e. the individual fusional vergence reserves.

## Introduction

Using two eyes has advantages for vision with respect to binocular summation and depth perception, but requires that the two eyes are properly coordinated on the oculomotor and sensory level. For a general review see Howard [1]. Conditions of non-optimal binocular coordination are not uncommon given the considerable individual differences and impairments that may appear as reduced stereo vision, low vergence functions, decompensated heterophoria, or asthenopic complaints; thus, optometric remediation is required [2, 3]. An important aspect of binocularity is the fixation disparity, which refers to conditions where the vergence angle between the two visual axes is not optimally adjusted and the retinal images of the two eyes are not optimally superimposed during the fusional process. There are two types of fixation disparity that differ in the measurement paradigm, the physiological meaning, and the area of applications. The classical clinical measure is the subjective fixation disparity [4], indicated by a perceived offset of physically aligned dichoptic nonius lines; precise nonius adjustments can be made in the range of some minutes of arc, also with simple test devices applied in clinical optometry [5–11]. Objective fixation disparity is a vergence error that is measured physically with eye tracker procedures; since the objective fixation disparity is typically below 1 degree, it can only be measured with sophisticated research methods that were mostly applied to simple fixation tasks [12–16]. More recently, natural viewing conditions included gaze shifts in space [17] and reading or scanning tasks [18–28]. Simultaneous recordings of subjective and objective fixation disparity are rare [12, 15, 29–33]. The present study seems to be the first to investigate whether subjective and objective fixation disparity are modified when observers wear prisms over several weeks in daily life which is one of the possible remediations of binocular impairments. The reasoning and hypotheses of this study result from the following physiological properties of the two types of fixation disparity.

**Fig 1:**
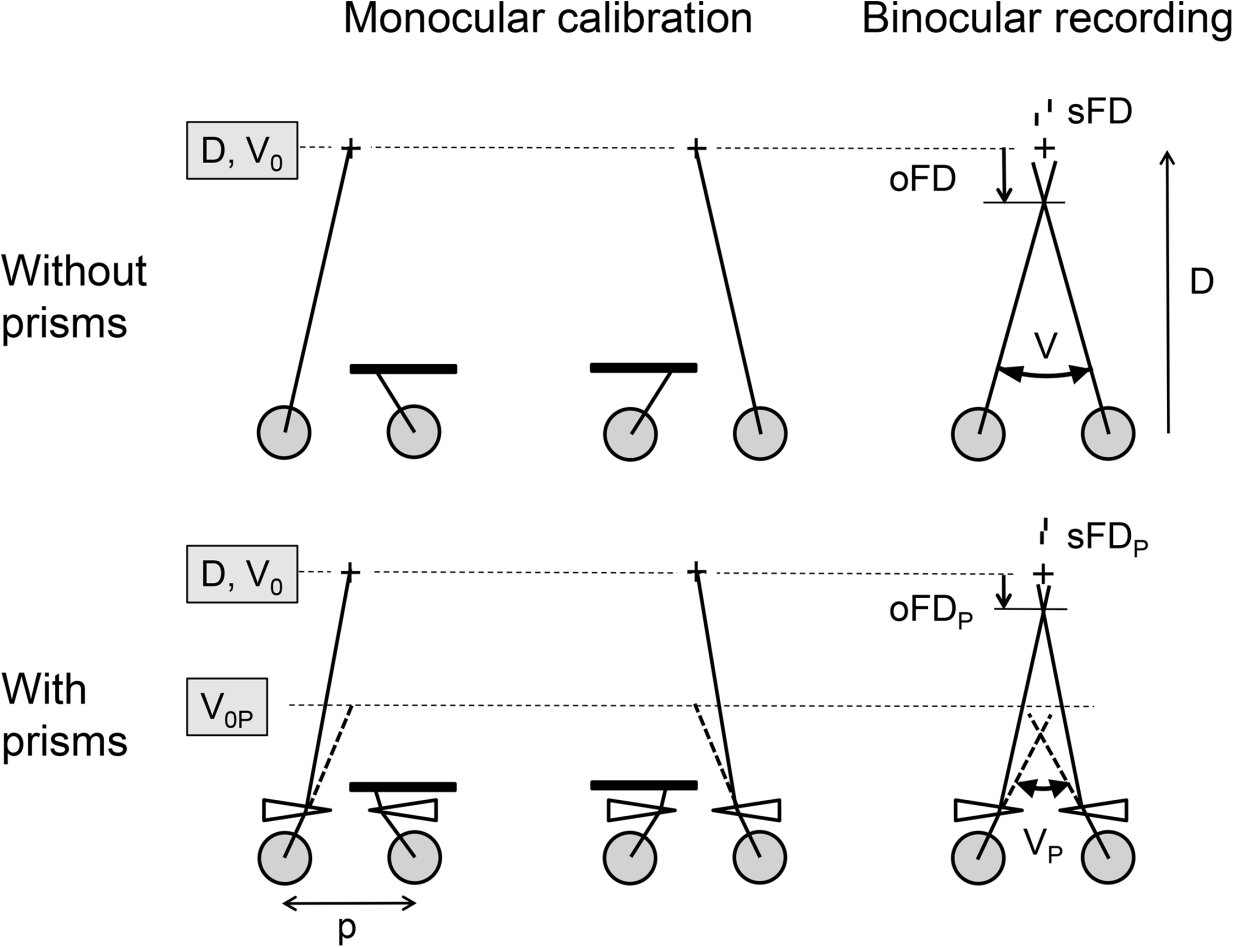
Conditions of measuring fixation disparity. Without prisms, the objective fixation disparity (oFD) is the difference between the observed vergence angle V during binocular recording and the stimulus vergence angle V_0_, which is geometrically given by the viewing distance D and the interpupillary distance p, i.e. V_0_ = 2 arctan (p/2D). In this example of an over-convergent (eso) oFD, V is larger than V_0_. The monocular components of V_0_ are measured during the eye tracker calibration that is made separately for the left and right eye: the eye position during monocular fixation represents the zero position for the subsequent binocular recording period. The covered eye assumes the heterophoria resting state. To correct an eso fixation disparity (as in this example), base-out prisms are applied. These prisms turn the visual axes optically outward (drawn lines), which requires the eye muscles to converge more (broken lines) to maintain fusion (see Fig 2). When prisms are applied, V_0P_ = Prismpower + V_0_ is the stimulus vergence angle and V_p_ is the vergence angle. The fixation disparity with prisms is measured relative to the reference condition of monocular fixation when the prisms are worn. The subjective fixation disparity (sFD = arctan (d_Non_/D)) is illustrated by the amount of nonius offset d_Non_, which is typically smaller than the objective fixation disparity. Note that both types of fixation disparity are smaller with prisms than without prisms, as suggested by the study results. The graphs show the case of visual axes that intersect in front of the fixation point; this over-convergent state is referred to as eso fixation disparity with a positive sign. In the opposite under-convergent state, the visual axes intersect behind the fixation point (exo fixation disparity with a negative sign); in the latter case, base-in prisms are applied.

The objective fixation disparity (oFD) refers to the oculomotor position of the eyes, i.e. the vergence angle V between the visual axes, which is measured with eye trackers using a monocular calibration procedure (Fig 1): targets fixated by the left or right eye alone are assumed to be projected onto the centre of the foveola and the corresponding left and right eye positions define a theoretical vergence angle V_0_, which is assumed to represent the optimal vergence state, i.e. zero objective fixation disparity (oFD = 0); thus monocular calibrations of the eye tracker define the reference for objective fixation disparity. Any deviating vergence state (vergence error) represents an objective fixation disparity which can be up to about 60 min arc. Nevertheless, double vision does not occur, rather the binocular targets appear as single objects, i.e. the left and right retinal images are fused and receive the same visual direction in space. Thus, despite of vergence errors, neural mechanisms with different stages provide sensory fusion of the binocular targets [1, 34]. However, even if the binocular stimuli are fused, two physically aligned monocular test stimuli for each eye (nonius lines) may nevertheless be perceived in different visual directions: this psychophysically measured nonius offset is referred to as subjective fixation disparity (sFD). A nonius test result is not a psychophysical equivalent of the oculomotor vergence error measured with eye trackers. Rather, the validity of nonius lines as vergence indicators is questionable [12, 35–37]. The subjective fixation disparity seems to be affected by two processes: the oculomotor adjustment of the visual axis (objective fixation disparity) and the mapping of visual directions by sensory fusion [38, 39]. The conditions of fusion play an important role. In non-fusion conditions, as in measurements of heterophoria or resting vergence (dark vergence), nonius lines give a very similar vergence angle as eye trackers [40, 41]. In fusion however, the visual directions in the vicinity of the binocular target are modified in a way to facilitate sensory fusion. This shift in retinal correspondence, however, can affect the visual directions of dichoptic nonius lines in the following way [1, 30, 31, 35, 38, 42]. When nonius lines are more distant from a local central fusion stimulus by about 4 deg, the subjective and objective fixation disparity are similar in amount. However, the closer the nonius lines are located near the fusion stimulus, the more the visual direction of the fusion stimulus is transferred to the nonius lines and the smaller is the subjective fixation disparity. Thus, the spatial arrangement of binocular and monocular objects in the test are important, but individuals differ in the effect of the gap between nonius lines and fusion object [43]. Some studies reported that objective fixation disparity can be 10 times larger than subjective fixation disparity, other studies found similar amounts (summarized in [33]). Given these physiological differences, both types of fixation disparity are only weakly correlated [33]. Still they have some common properties: they both are correlated with the resting vergence position, measured as heterophoria or dark vergence [20, 21, 33, 44]; they both increase as the viewing distance shortens [13, 44].

**Fig 2:**
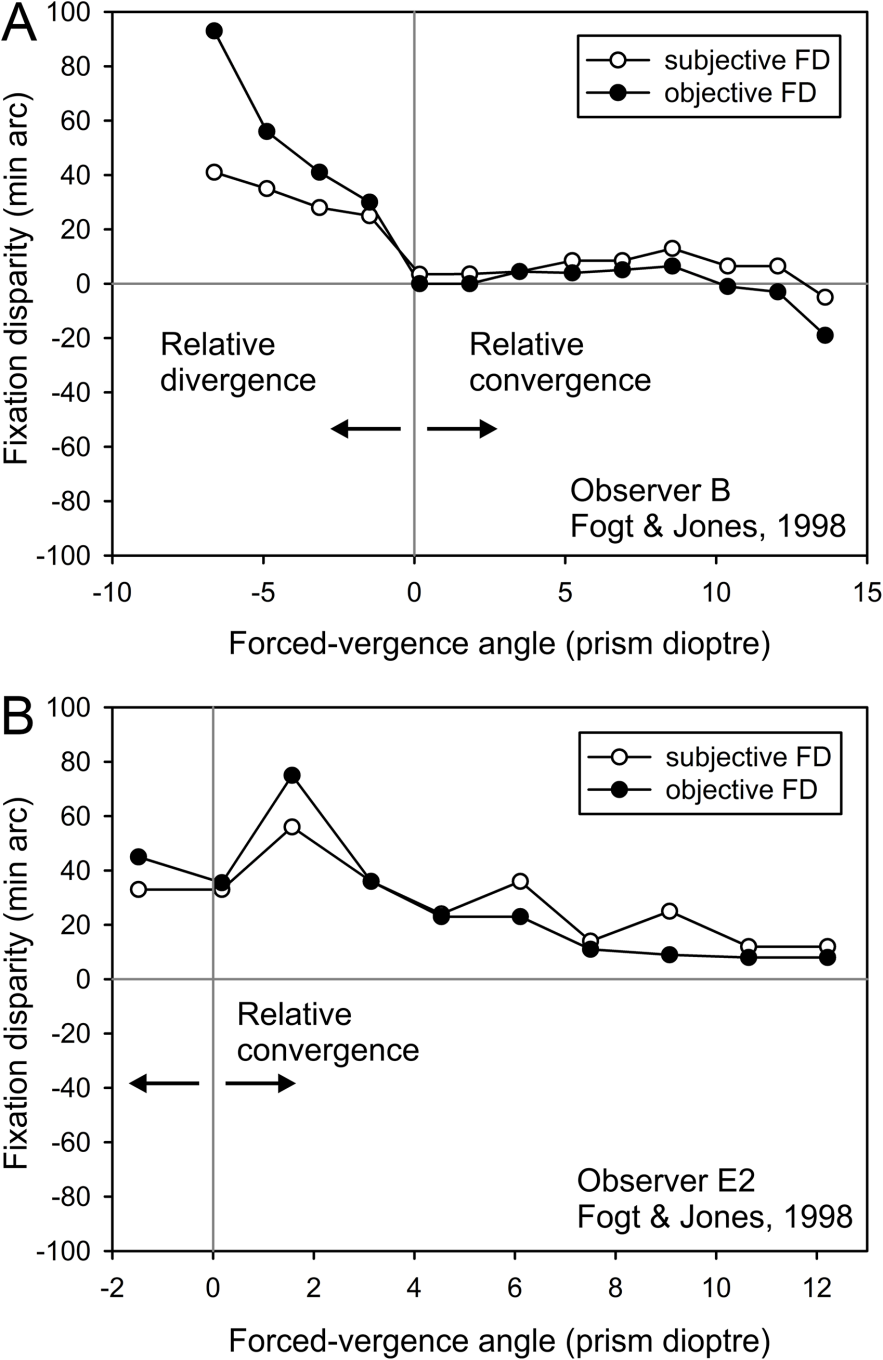
Forced-vergence fixation disparity curves, replotted from Fogt and Jones [30]. Subjective and objective fixation disparity (sFD and oFD) are measured as a function of the forced-vergence angle for two individuals (B and E2). The zero position on the x-axis represents the baseline vergence angle, corresponding to the viewing distance of 73 cm in [30]. Relative to this baseline, the vergence stimulus is varied in the convergent (positive) and divergent (negative) direction, corresponding to base-out and base-in prisms, respectively. For fixation disparity, positive and negative values mean an over-convergence (eso) or under-convergence (exo), respectively.

The fixation disparity can be modified by varying the vergence stimulus, i.e. the absolute disparity of the two retinal images which can technically be made by a lateral displacement of the monocular images in a laboratory stereoscope [29] or by prisms in front of the eyes in optometry [6]; in natural vision, fixation disparity varies with viewing distance [44]. The physiological mechanism of correcting a fixation disparity is described by the so-called “forced-vergence fixation disparity curves”, as illustrated in Fig 2, which includes replotted data of two individuals in the study of Fogt and Jones [30]. “Forced-vergence” is understood as a change in absolute disparity of a fusion stimulus, while the accommodative stimulus (i.e. the viewing distance) remains unchanged. The zero position on the x-axis in Fig 2 represents the baseline vergence angle corresponding to the physical viewing distance. Relative to this, increasing forced-convergence (forced-divergence) of the ocular muscles leads to an exo (eso) shift in fixation disparity. E.g. in a case with an eso fixation disparity (Fig 2B) forced convergence can shift the fixation disparity in the exo direction. More generally, the typically negative slope of forced-vergence fixation disparity curves means that in order to reduce an eso (exo) fixation disparity, the eye muscles have to exert a relative convergence (divergence): this is reached by the application of base-out (base-in) prisms that optically turn the visual axes outward (inward); see Fig 1. In other words, the prism-induced vergence change (response) is smaller than the amount of the prism (stimulus), so that the effective fixation disparity is reduced. Also when prisms are applied, monocular calibrations of the eye tracker define the reference for objective fixation disparity with prisms, i.e. oFD_p_ (see Fig 1). Fixation disparity curves are measured subjectively in clinical optometry with dichoptic nonius lines [5, 6, 11, 45, 46]. Measurements of objective fixation disparity curves are confined to the research laboratory since elaborate eye tracking facilities are required [12, 15, 30, 31, 35, 42]. Fogt and Jones [30] found a tendency of steeper curves in objective than in subjective fixation disparity (see Fig 2).

Laboratory and clinical optometric research showed that a fixation disparity reflects a non-optimal vergence state and that prisms may have a benefit. First, there is considerable evidence from application of the Mallett unit [47, 48] that an exo subjective fixation disparity in near vision (or a base-in aligning prism required to compensate a subjective fixation disparity) is associated with asthenopic symptoms [49–52]. A base-in aligning prism at near is associated with reduced fusional vergence reserves in the opposing direction [53]. Second, studies based on the Mallett-unit showed that an artificially induced fixation disparity (by means of prisms) can reduce binocular acuity [54]. An ergonomic approach showed that particularly those observers with a large exo fixation disparity in near vision preferred a computer monitor at a longer viewing distance where fixation disparity is smaller [55, 56]. An optometric remediation of a fixation disparity is constantly wearing appropriate prisms and there are several studies on possible effects. Prism corrections based on the near Mallett unit improved binocular visual acuity, particularly with base-in prisms [57]; further, these prisms were preferred for everyday use [58] and improved the reading rate [59]. Presbyopic subjects with convergence insufficiency reported fewer symptoms when wearing base-in prisms, and the effect tended to depend on the pre-treatment heterophoria and near point of convergence [60, 61]. In asthenopic subjects, base-in prisms of 4 prism dioptre improved the binocular acuity [62]. The “Measuring and Correcting Methodology after H.-J. Haase (MCH)” [63–67] determines prisms primarily from far vision tests to achieve a more normal resting vergence position. While the hypothetical assumptions of this procedures have been challenged [68–71], the benefit of wearing these prisms had been shown in results of clinical vergence measures [72, 73] and contrast sensitivity [74]. Effects of MCH-prisms have been compared with prisms prescribed from orthoptic examinations [75].

However, when prisms are constantly worn, the adaptability of the vergence system may play a role [76–83]. First, this is reflected in the slope of the fixation disparity curves: flat or steep curves, respectively, indicate a more or less adaptive vergence system which tends to be associated with less or more asthenopic complaints [11]. Fixation disparity curves are typically measured with a rather short exposure of the prisms (e.g. 15 s) in order to minimize vergence adaptation [3]. However, if prisms are used in eyeglasses for constant wear, the vergence system may (partly) adapt to this forced-vergence state and the intended reduction in fixation disparity may disappear, or be smaller than intended [45].

The present study seems to be the first to measure the effect of 5 weeks of wearing prisms on objective and subjective fixation disparity, in order to test their specific responses to wearing prisms. The hypotheses result from the typical form of forced-vergence fixation disparity curves (see Fig 1 and Fig 2). To correct for an eso fixation, base-out prisms are applied that force the eye muscles to converge which should shift the fixation disparity into the exo direction. An exo fixation disparity requires base-in prisms that force the eye muscles to diverge and should shift the fixation disparity into the eso direction. Given the different directions of these effects, subgroups receiving base-in and base-out prisms were analysed separately. Since we measured objective and subjective fixation disparity simultaneously, we investigated relations between these two types of fixation disparity and between prism effects in these both dependent variables. The prior general hypothesis would be that prism effects should resemble in objective and subjective fixation disparity. As modulating variable we tested the effect of the adaptability of the vergence system, using the fusional vergence reserve as an individual optometric parameter.

Additionally, the nonius bias was measured, i.e. the offset required for perceived alignment of binocularly presented nonius lines. The nonius bias is correlated with subjective fixation disparity [84, 85] so that the question arises whether the nonius bias may change by wearing prisms. Further, since there is evidence [69] that the objective vergence position may be influenced by the dichoptic nature of nonius lines, we compare the objective fixation disparity when nonius lines are presented binocularly or dichoptically. These results are reported in the Supporting Information (S1 Text).

## Materials and Methods

### Design of the study

The study comprised the following steps:

1. Refraction, optometric tests, and prism prescription
2. Fixation disparity measures without prisms (two measurements within 2 weeks)
3. Wearing the prism eyeglasses for about 5 weeks
4. Fixation disparity measures with prisms (two measurements within 2 weeks)

### The participants: refraction, optometric tests, and prism prescription

Participants were students or staff of the University of Applied Sciences and Arts Northwestern Switzerland, but without optometric knowledge. They were paid for voluntary participation and were informed about the intention of the study to investigate the potential effect of prism eyeglasses on visual functions. After the study, they were allowed to keep the eyeglasses. All procedures were approved by the Ethic Board of the Leibniz Research Centre of Working Environment and Human Factors (IfADo, Dortmund, Germany) and were in accordance with the declaration of Helsinki. The prescription of prism eyeglasses was according to the regulations of the European Diploma of the European Council of Optometry and Optics (http://www.ecoo.info/) and according to the code of conduct of the World Council of Optometry (http://www.worldoptometry.org/en/about-wco/code-of-conduct.cfm). Participants gave their written consent after being informed about the procedures.

24 participants were recruited to meet the following the criteria: emmetropia (not wearing glasses nor contact lenses), no latent hyperopia, visual acuity in far and near distance sc ≥ 1.0, with a difference in visual acuity between the two eyes less than 1 logMAR line, TNO stereo thresholds < 120”, no strabism, no eye disease. For the anamnesis of astenopia, the “Convergence Insufficiency Symptom Survey” was applied [86]. The anterior and posterior segments of each eye were inspected, including van Herick estimation of the chamber angle [87]. An objective refraction was made [88] and noncontact intraocular pressure was measured. Mean ± SD of age was 23.5 ± 2.8 years, ranging from 18 to 30 years (6 female, 18 male).

Table 1 describes refractions and the prisms prescribed. The sample was formed to have two subgroups of 12 participants with the two directions of prisms. The group with base-out prisms had a mean ± SD dissociated phoria (van Graefe) of 0.7 ± 2.0 Δ base-out; fusional vergence reserves (far distance) had a break point of 16.0 ± 7.0 Δ and a recovery point of 8.6 ± 2.6 Δ in the base-out direction and corresponding points of 7.4 ± 2.5 Δ and 2.9 ± 1.4 Δ in the base-in direction. The group with base-in prisms had a dissociated phoria of—0.6 ± 1.9 Δ base-in; fusional vergence reserves (far distance) had a break point of 10.6 ± 4.1 Δ and a recovery point of 8.6 ± 2.6 Δ in the base-out direction and corresponding points of 6.5 ± 2.3 Δ and 3.5 ± 1.4 Δ in the base-in direction. The mean “Convergence Insufficiency Symptoms Score” [86] was 11.8 ± 6.2 in the base-out group and 10.4 ± 6.6 in the base-in group which indicates that both groups can be considered as asymptomatic since the score is below the cut-off level of 21.

**Table 1:** Refractions and prisms for the 24 participants. BO and BI refers to prisms base-out and base-in, BU and BD refers to prisms base-up and base-down.

	Right eye					Left eye					Both eyes
Subject	Sph (D)	Cyl (D)	Axis (deg)	Prism (Prism dioptre, Δ)		Sph (D)	Cyl (D)	Axis (deg)	Prism (Prism dioptre, Δ)		Horizontal prism
	**Base-out prisms**										
**01**	+0.25	-0.25	175	4.00 BO	0.25 BU	+0.25			4.00 BO	0.5 BD	8.00 BO
**02**	-0.50	-0.25	25	3.25 BO	0.25 BU	-0.50	-0.25	30	3.25 BO		6.50 BO
**03**	-0.25	-0.25	170	3.00 BO		-0.50	-0.25	85	3.00 BO		6.00 BO
**04**	0.00	-0.25	10	2.05 BO		0.00	-0.25	168	2.50 BO		5.00 BO
**05**	+0.25	-0.25	85	1.75 BO		-0.25			1.75 BO	0.5 BU	3.50 BO
**06**	+0.25			1.75 BO	0.50 BD	+0.25	-0.50	13	1.75 BO		3.50 BO
**07**	+0.50			1.50 BO	0.25 BU	+0.50			1.75 BO		3.25 BO
**08**	+0.25	-0.75	90	1.50 BO	0.25 BU	+0.25	-0.75	71	1.50 BO	0.5 BD	3.00 BO
**09**	+0.50	-0.50	136	1.50 BO	0.25 BD	+0.50	-0.50	55	1.50 BO		3.00 BO
**10**	+0.50	-0.25	168	1.25 BO	0.25 BD	+0.50	-0.25	19	1.25 BO		2.50 BO
**11**	+0.25	-0.50	108	0.75 BO		0.00			0.75 BO	0.25 BD	1.50 BO
**12**	0.00	-0.50	9	0.75 BO		-0.25	-0.50	0	0.75 BO		1.50 BO
	**Base-in prisms**										
**13**	0.00	-0.50	85	- 0.50 BI		+0.75	-0.75	104	- 0.50 BI	0.25 BD	- 1.00 BI
**14**	0.00			- 0.75 BI	0.50 BU	0.00			- 0.75 BI	0.5 BD	- 1.50 BI
**15**	0.00	-0.25	88	- 1.00 BI		0.00	-0.25	86	- 1.00 BI	0.25 BD	- 2.00 BI
**16**	0.00	-0.50	91	- 1.00 BI	1.00 BU	+0.25			- 1.00 BI	1.25 BD	- 2.00 BI
**17**	+0.50			- 1.25 BI	0.50 BD	+0.50			- 1.25 BI	0.5 BU	- 2.50 BI
**18**	+0.50			- 1.50 BI		+0.50	-0.25	64	- 1.50 BI		- 3.00 BI
**19**	-0.50			- 1.50 BI	0.50 BD	-0.25	-0.25	100	- 1.50 BI		- 3.00 BI
**20**	-0.25	-0.25	168	- 1.50 BI	0.50 BU	-0.25	-0.25	178	- 1.75 BI	0.25 BD	- 3.25 BI
**21**	0.00	-0.50	95	- 1.75 BI		-0.25	-0.25	70	- 1.75 BI		- 3.50 BI
**22**	+0.50			- 2.25 BI	0.25 BU	+0.25	-0.25	160	- 2.25 BI		- 4.50 BI
**23**	+0.50	-0.25	104	- 2.50 BI		+0.50	-0.25	16	- 2.25 BI		- 4.75 BI
**24**	+0.25			- 2.75 BI	0.25 BU	+0.25	-0.25	2	- 2.75 BI		- 5.50 BI

**Fig 3:**
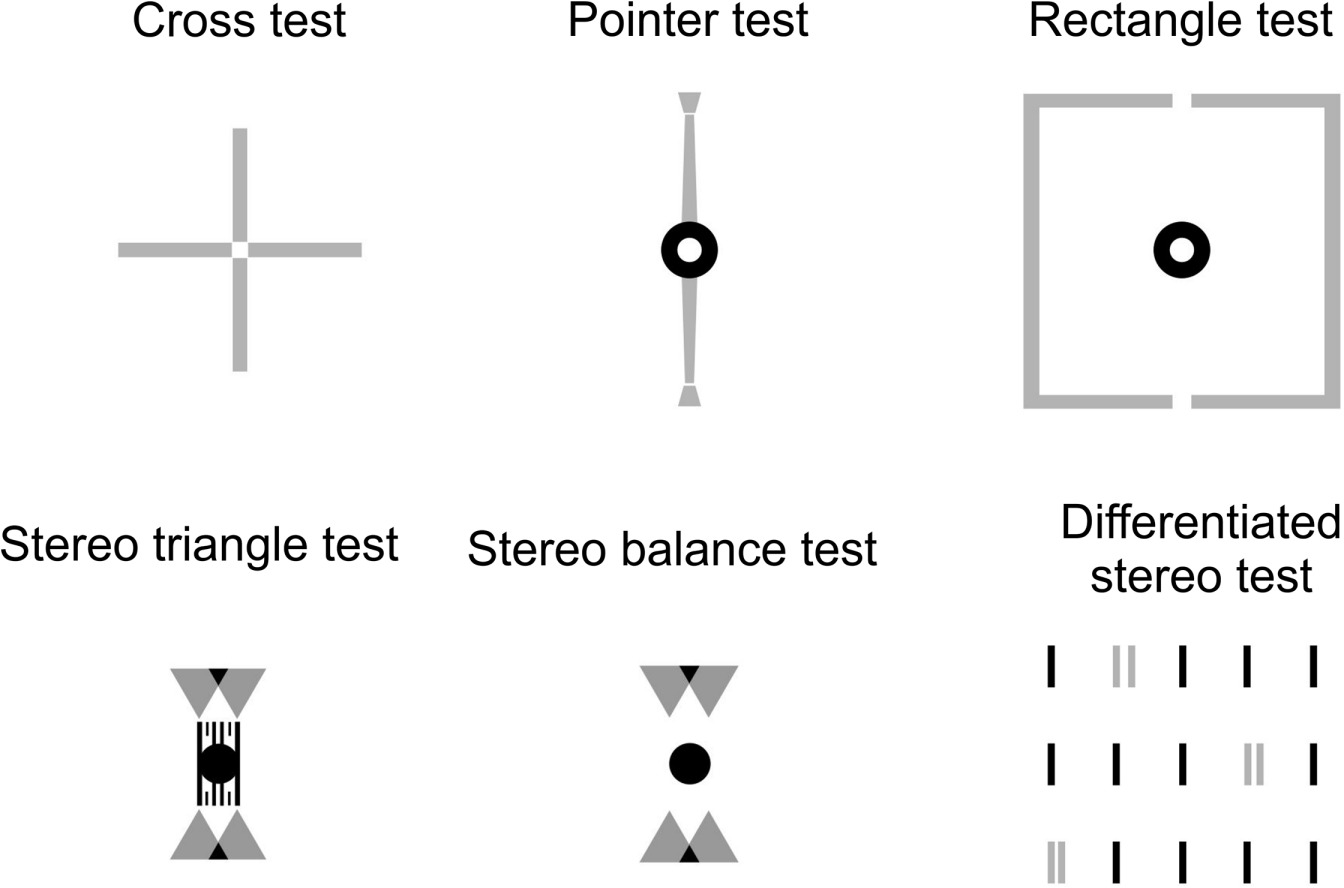
The MCH-method for prescribing prisms. Six different test plates are applied at a 6 m viewing distance. Black parts are binocularly visible fusion targets, grey parts are presented dichoptically (i.e., separately to the two eyes). For scale: each of the four lines in the Cross test is 1.2 deg long.

The prism prescription followed the detailed “Guidelines for the application of the Measuring and Correcting Methodology after H.-J. Haase”(MCH) [89]. With the correction of subjective refraction in the trial frame, a series of test plates were presented (Cross test, Pointer test, Rectangle test, Stereo triangle test, Stereo balance test, Differentiated stereo test; see Fig 3) and the prism power required for perceived alignment of the dichoptic markers was modified from one test to the other in order to finally reach subjective alignment of dichoptic targets in all tests and to achieve best stereo vision in the crossed and uncrossed direction. For the prism eyeglasses, the lens type Hoya HiLux 1,6 Clean Vision was used.

### Test stimuli for subjective and objective fixation disparity

**Fig 4:**
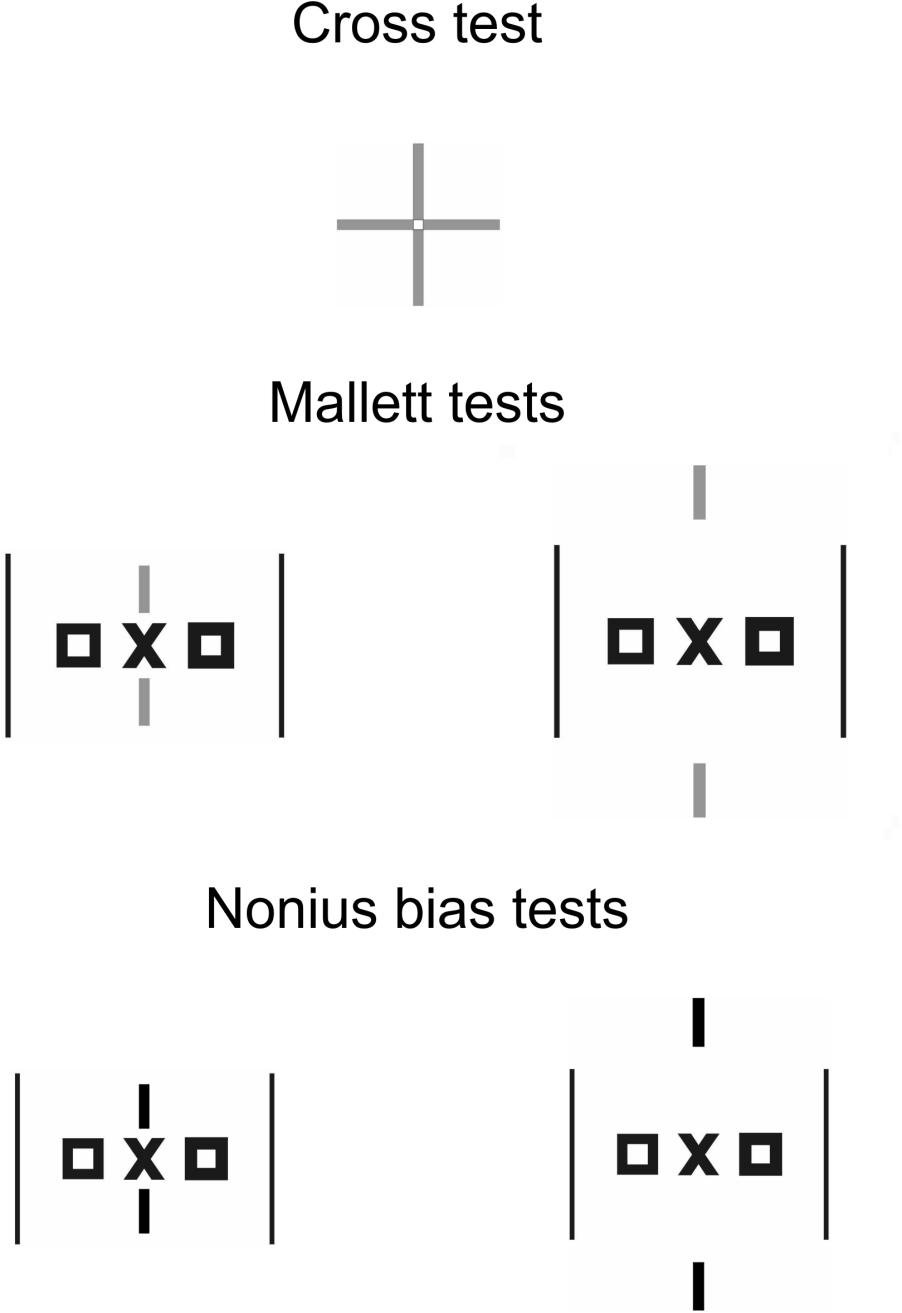
Testing of the dependent variables. The three tests (Cross test, Mallett tests and Nonius bias tests) were used for measuring subjective and objective fixation disparity, without and with prisms, respectively. For scale: each of the four lines in the Cross test is 1.2 deg long.

Fig 4 shows the test stimuli for subjective and objective fixation disparity, which were the dependent variables. They were presented at a 6 m viewing distance on a 3D monitor using circular polarization (LG 32 LW 4500); the screen area was 6.6 deg x 3.7 deg. The luminance was 73 cd/m^2^ on the test field, 24 cd/m^2^ on the outer frame of the monitor and 44 cd/m^2^ on the surrounding walls.

The test stimuli differed in the spatial structure of fusional contours and dichoptic nonius targets (see Fig 4). All stimuli appeared within a white field square of 2.8 deg on the dark screen background. The Cross test belongs to the series of tests in the MCH-test procedure (see above) and does not include a central fusion stimulus, rather vertical lines are presented to the right eye and horizontal lines to the left eye (each of the four lines is 1.2 deg long); fusion is stimulated more peripherally by the borders of the 2.8 deg white square. Stimuli without central fusion objects were also used in previous research [5, 6, 11, 30, 31] and these tests tend to show a larger and more variable subjective fixation disparity [90, 91] than tests like the Mallett test [47, 48], which include a central fusion object (OXO), adjacent dichoptic vertical nonius lines and more peripheral fusion objects. The central fusion object better resembles a condition of normal viewing in which central fixation target typically occur. The Mallett type of test was used in two versions: with nonius lines closer and more distant to the central fusion target. The third type of test resembles the Mallett test, except that the nonius lines are not presented dichoptically, but are visible by both eyes; this arrangement does not measure a vergence state, but the nonius bias, i.e. a perceived misalignment of fused nonius lines that have a certain vertical separation [84, 85]. The Nonius bias test was also used in two versions with different amounts of vertical nonius separation.

### Psychophysical method for subjective fixation disparity and nonius bias

For the subjective measures, the tests included two nonius targets that were continuously visible (see Fig 4). The nonius targets appeared with a random offset; subsequently, the observer shifted the targets relative to each other using the left and right computer mouse button and indicated perceived alignment by clicking the centre mouse button. Immediately, the nonius targets reappeared with an arbitrary offset again and the observer started the next adjustment. In this way, a series of adjustments was made within the one minute recording period; thus, the number of adjustments depended on the individual speed of adjustment. From one adjustment to the next, the white quadratic test pattern was shifted by about 2.5 deg from left to right on the rectangular display. This required small saccades between the adjustments and was used to prevent a continuous static fixation which is rather unnatural and stressfull.

Both subjective fixation disparity (sFD) and nonius bias (NB) were calculated from the adjusted nonius offset d_Non_ and the viewing distance D (6m) [sFD (or NB) = arctan (d_Non_/D)]. Positive values refer to an eso fixation disparity or a nonius bias with the upper line left of the lower line when perceived in coincidence; negative values refer to the opposite cases. This nonius technique is able to detect small mean changes in the order of 0.5 min arc, both in studies using within-subjects and between-subjects designs [9, 10, 56, 92].

### Eye movement recording for objective fixation disparity

We measured eye movements with the video-based EyeLink II (SR Research Ltd, Osgoode ON, Canada), using the dark pupil system that tracks the centre of the pupil. We modified the conventional EyeLink modes of operation in order to improve performance for measuring objective fixation disparity [33]. We used a chin and forehead rest, a band around the head and narrow temporal rests to minimize artifacts due to possible lateral and oblique head movements; a bite bar was not used. The cameras have been fixed to the head rest. A polarized goggle was always in front of the eyes for the dichoptic separation of the nonius lines, prism eyeglasses were worn in the post-tests; the polarized goggles and the eyeglasses lenses were installed a few centimetres away from the eyes so that the EyeLink cameras could be installed below to have a free unobstructed view of the eyes.

Instead of the original EyeLink II calibration mode, we used the raw data and applied purpose-made calibrations before and after the one minute recording period, the average of both calibrations was used. For the monocular calibration, subjects were requested to carefully fixate one of nine calibration targets (crosses of 2 min arc) that appeared sequentially in the screen centre and deviating positions at 187 min arc horizontally and 98 min arc vertically. In order to achieve monocular vision during calibration, the left eye was covered with an opal occluder for viewing the series of the nine targets with the right eye and, subsequently, the right eye was covered for viewing the nine targets with the left eye. For each eye, the target presentations had a random order. Recorded data were analyzed based on the raw data—sampled at a rate of 2 ms (500 Hz). For the calibration calculation, we used the MATLAB procedure regrMLR.M, which provides a 2-dimensional robost linear regression.

The measurement paradigm and definition of objective fixation disparity is illustrated in Fig 1. The measurement comprised two steps: (a) The determination of the monocular reference eye positions in the calibration phase, i.e. the gaze direction for each eye in monocular fixations. This corresponds to the geometrically expected vergence angle V_0_ = 2 arctan (p/2D) depending on the individual interpupillary distance, i.e. V_0_ = 0.6 deg for the viewing distance of 6 m and a typical 6.3 cm interpupillary distance. (b) The recording of the vergence angle V from the left and right eye signal of the eye tracker. With and without prisms the reference condition is the monocular fixation when the target is assumed to be projected onto the centre of the foveola, i.e. corresponding to the principle visual direction.

The amount of objective fixation disparity is typically below 1 deg, which is a challenge for video-based eye trackers. Therefore, all procedures were optimized for the present purpose [33]. The noise level of the EyeLink system is below 1 min arc, meaning that such small changes in eye position can physically be detected. In principle, a fixation disparity is a change in vergence when the test condition changes from monocular to binocular viewing, i.e. all vergence measures are calculated in relation to a monocular reference condition during the calibration. To avoid instabilities over longer test periods, other research used short-term changes within a few seconds between monocular and binocular vision. This, however, also introduces adaptive processes between these two conditions, so that a “direct procedure” has been suggested as more favorable [16], i.e. monocular calibrations before/after longer binocular periods. As a compromise between short recordings for avoiding instabilities and longer recordings to collect more data (also for a series of adjustments for the subjective measures) we chose a one minute recording period. Moreover, averaging across a series of recorded data and repeated sessions reduces random influences of measurement errors.

We specifically addressed a potential artifact of video eye trackers due to variations in the pupil size that may occur in the course of experimental runs or between conditions. Following Yang et al. [93] and Camellin et al. [94], a dilatation of the pupil by 1 mm produces a temporal (outward) shift of the centre of the pupil by about 0.05 mm, on the average. This means that a 1 mm increase in pupil diameter would appear as a more exo fixation disparity by about 30 min arc assuming a 12 mm radius of the eye, as calculated from dFD = 2 *arc tan (0.05mm/12mm) for the pair of the two eyes. To account for such possible effects, we recorded the pupil diameter expressed in subpixels within the image of the eyes provided by the EyeLink system. A conversion to real pupil size depends on the actual position of the camera relative to the eye which we sometimes needed to adjust between different observers and runs in these experiments. Such individual and case-by-case calibration of the pupil with an object of known size was not made. However, we converted the group average pupil size in subpixel values to millimeters based on an average calibration known from pilot tests in our laboratory. This procedure is based on the assumption that in groups of observers the calibration factor may be similar. At least, we were able to analyze whether the group mean pupil size varies or remains constant. The pupil size was not expected to depend on wearing prisms beforehand, but was analyzed since pupil variations are a possible artifact in video eye tracking methods. Results are reported in the Supporting Information (S2 Text).

### Procedure of measurement

**Fig 5:**
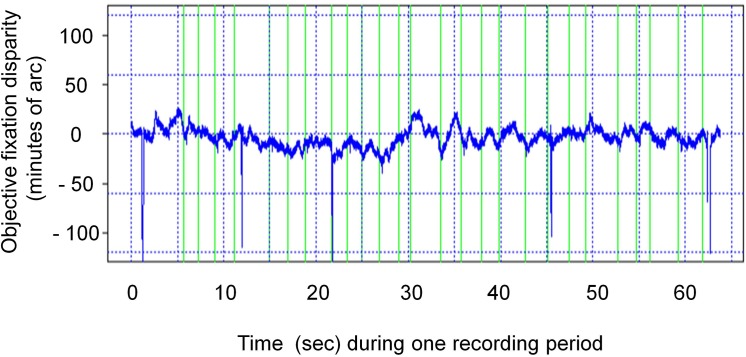
Example of a 1 minute recording period of fixation disparity. The 22 green markers indicate when the observer had finished shifting the nonius targets to perceived alignment which represents a measure of subjective fixation disparity. At the same moments in time, the objective fixation disparity was sampled from the continuous stream of data (blue trace). This procedure provides simultaneous recordings of both types of fixation disparity. Regressions across the sampled data allow analysing the time trend within the 60 second recording period.

Each single block of recording had three phases: the pre-calibration of the eye tracker, the one minute test phase, and the post-calibration. During the one minute test phase, a series of nonius adjustments was made and simultaneous samples of objective eye movement measures were taken (Fig 5). The order of these blocks was balanced across the test patterns: half the sample had the sequence Cross test, Mallett tests, Nonius bias tests, the other half had the reversed sequence. Without wearing prisms, two sessions were made, separated by about one week. After wearing the prisms for whole days over a period of 35 ± 9 days (mean ± SD), two sessions were made while participants wore the prisms.

### Statistical analyses

A series of stratified data analyses (i.e. on subsets of data and samples) were conducted, in order to separately address specific optometric hypotheses, as different effects of base-in and base-out prisms (due to the typically non-symmetrical prism fixation disparity curves), or specific effects in different types of tests (due to their different fusion conditions). This stratification avoids difficulties in interpretations that could arise in a full complex statistical model, where a larger number of factors and interactions would be included.

For a concise description of results, the data of the two repeated sessions were averaged, both with and without wearing prisms. Further, the two versions of the Mallett test and of the Nonius bias test (with different vertical nonius separation) were also averaged, since they revealed similar results.

In this way, the two dependent variables (objective and subjective fixation disparity) were analyzed with linear mixed effects models with three factors (see Table 2): (1) prism versus no prisms, (2) individual amount of prism, (3) time effects within the 60 second recording period, using ln(time(s)/1s) as ln-transformed time scale where the resulting coefficient gives the change within 2.7 s. The interaction (prism/no prism) x (individual amount of prism) was tested to see whether the amount of the individual prism may modulate the effects. The estimated pupil diameter was analyzed in the same way and results are described in the Supporting Information (S2 Text) to quantify the extent to which the measure of objective fixation disparity may be affected by possible variation in pupil diameter. The function lme of the open-source statistical software R was used [95, 96].

**Table 2:** Results of the statistical linear mixed effects model, separately for (1) the objective and subjective measures and (2) the Cross test, Mallett tests, and Nonius bias tests.

Objective measure: fixation disparity (oFD)									Subjective measures: fixation disparity (sFD) and Nonius bias (NB)								
oFD	Test	Base	Coefficient	Value (minarc)	Std. Error (minarc)	DF	t-value	p-value	sFD(NB)	Test	Base	Coefficient	Value (minarc)	Std. Error (minarc)	DF	t-value	p-value
**oFD**	**Cross**	**Base**	(Intercept)	25.93	5.61	633	4.63	0.000	**sFD**	**Cross**	**Base**	(Intercept)	-2.38	0.86	633	-2.77	0.006
	**test**	**in**	**Prism effect (Cohen’s d = -0.25)**	**-0.84**	**1.67**	**633**	**-0.50**	**0.615**		**test**	**in**	**Prism effect (Cohen’s d = 1.09)**	**2.52**	**0.26**	**633**	**9.82**	**0.000**
			Amount of prism	3.77	2.79	10	1.35	0.206				Amount of prism	0.80	0.43	10	1.88	0.090
			ln(time(s)/1s)	-8.17	1.25	633	-6.53	0.000				ln(time(s)/1s)	0.07	0.19	633	0.35	0.724
			Prism effect: Amount of prism	-1.48	1.32	633	-1.13	0.261				Prism effect: Amount of prism	-0.14	0.20	633	-0.67	0.501
**oFD**	**Cross**	**Base**	(Intercept)	53.67	7.03	654	7.63	0.000	**sFD**	**Cross**	**Base**	(Intercept)	2.56	1.27	654	2.01	0.045
	**test**	**out**	**Prism effect (Cohen’s d = -0.69)**	**-19.79**	**1.61**	**654**	**-12.32**	**0.000**		**test**	**out**	**Prism effect (Cohen’s d = -0.23)**	**-0.33**	**0.20**	**654**	**-1.63**	**0.104**
			Amount of prism	3.25	3.09	10	1.05	0.317				Amount of prism	0.80	0.61	10	1.31	0.218
			ln(time(s)/1s)	-6.83	1.11	654	-6.13	0.000				ln(time(s)/1s)	0.72	0.14	654	5.15	0.000
			Prism effect: Amount of prism	4.45	0.93	654	4.77	0.000				Prism effect: Amount of prism	-0.22	0.12	654	-1.90	0.058
**oFD**	**Mallett**	**Base**	(Intercept)	27.77	4.16	1335	6.68	0.000	**sFD**	**Mallett**	**Base**	(Intercept)	0.17	0.44	1335	0.38	0.703
	**tests**	**in**	**Prism effect (Cohen’s d = 0.42)**	**6.08**	**1.12**	**1335**	**5.42**	**0.000**		**tests**	**in**	**Prism effect (Cohen’s d = 0.46)**	**0.70**	**0.09**	**1335**	**7.65**	**0.000**
			Amount of prism	0.40	2.34	10	0.17	0.868				Amount of prism	0.62	0.28	10	2.18	0.054
			ln(time(s)/1s)	-7.87	0.83	1335	-9.46	0.000				ln(time(s)/1s)	0.04	0.07	1335	0.53	0.597
			Prism effect: Amount of prism	5.69	0.86	1335	6.62	0.000				Prism effect: Amount of prism	-0.25	0.07	1335	-3.63	0.000
**oFD**	**Mallett**	**Base**	(Intercept)	46.37	4.77	1413	9.72	0.000	**sFD**	**Mallett**	**Base**	(Intercept)	2.56	0.75	1413	3.44	0.001
	**tests**	**out**	**Prism effect (Cohen’s d = -0.53)**	**-10.41**	**1.11**	**1413**	**-9.39**	**0.000**		**tests**	**out**	**Prism effect (Cohen’s d = -0.23)**	**-0.34**	**0.12**	**1413**	**-2.91**	**0.004**
			Amount of prism	2.71	2.06	10	1.31	0.218				Amount of prism	0.01	0.36	10	0.03	0.973
			ln(time(s)/1s)	-6.70	0.77	1413	-8.70	0.000				ln(time(s)/1s)	0.18	0.08	1413	2.21	0.027
			Prism effect: Amount of prism	2.65	0.59	1413	4.46	0.000				Prism effect: Amount of prism	0.23	0.06	1413	3.77	0.000
**oFD**	**Nonius**	**Base**	(Intercept)	46.90	4.23	1412	11.08	0.000	**NB**	**Nonius**	**Base**	(Intercept)	0.21	0.21	1412	1.00	0.318
	**bias**	**in**	**Prism effect (Cohen’s d = 0.37)**	**5.35**	**1.12**	**1412**	**4.79**	**0.000**		**bias**	**in**	**Prism effect (Cohen’s d = -0.05)**	**-0.04**	**0.06**	**1412**	**-0.61**	**0.541**
	**tests**		Amount of prism	4.38	2.50	10	1.75	0.111		**tests**		Amount of prism	0.18	0.11	10	1.59	0.142
			ln(time(s)/1s)	-10.52	0.80	1412	-13.11	0.000				ln(time(s)/1s)	-0.19	0.04	1412	-4.26	0.000
			Prism effect: Amount of prism	3.97	0.87	1412	4.58	0.000				Prism effect: Amount of prism	0.04	0.05	1412	0.89	0.376
**oFD**	**Nonius**	**Base**	(Intercept)	50.92	3.89	1534	13.08	0.000	**NB**	**Nonius**	**Base**	(Intercept)	0.27	0.18	1534	1.49	0.138
	**bias**	**out**	**Prism effect (Cohen’s d = -0.17)**	**-3.74**	**1.05**	**1534**	**-3.56**	**0.000**		**bias**	**out**	**Prism effect (Cohen’s d = -0.55)**	**-0.29**	**0.06**	**1534**	**-4.93**	**0.000**
	**tests**		Amount of prism	6.82	1.59	10	4.29	0.002		**tests**		Amount of prism	-0.05	0.06	10	-0.77	0.460
			ln(time(s)/1s)	-8.37	0.71	1534	-11.76	0.000				ln(time(s)/1s)	-0.06	0.04	1534	-1.62	0.104
			Prism effect: Amount of prism	-3.75	0.53	1534	-7.04	0.000				Prism effect: Amount of prism	-0.06	0.03	1534	-1.90	0.057

For calculating effect sizes of fixation disparity, the difference between the prism conditions was divided by the pooled inter-individual standard deviation of the sample and test condition; this was made for group mean effects (and gives the conventional Cohen’s effect size d [97]) and was also made for individual effects (see below). This procedure indicates the changes in fixation disparity in relation to the distribution of fixation disparity. Effect sizes of 0.5 or 1.0 are interpreted as medium or large, respectively.

Regressions and correlations were calculated for testing the relation between individual measures. Given the small sample that may include potential outliers, the procedure “lmrob” from the package "robustbase" for the software R [95] was used for calculating robust estimations of regression lines, correlation coefficients and levels of significance. One-tailed p-values were only used when clear directional hypotheses apply.

## Results

The results comprise three steps. First, group mean results are presented. Second, relations between sensory and motor effects are analysed by correlations between individual prism effect sizes in subjective versus objective fixation disparity. Third, the individual response patterns are compared with the fusional vergence reserve to test influences of vergence adaptation. The Supporting Information includes results on the nonius bias and the pupil diameter.

### Mean results of fixation disparity with and without prisms

**Fig 6:**
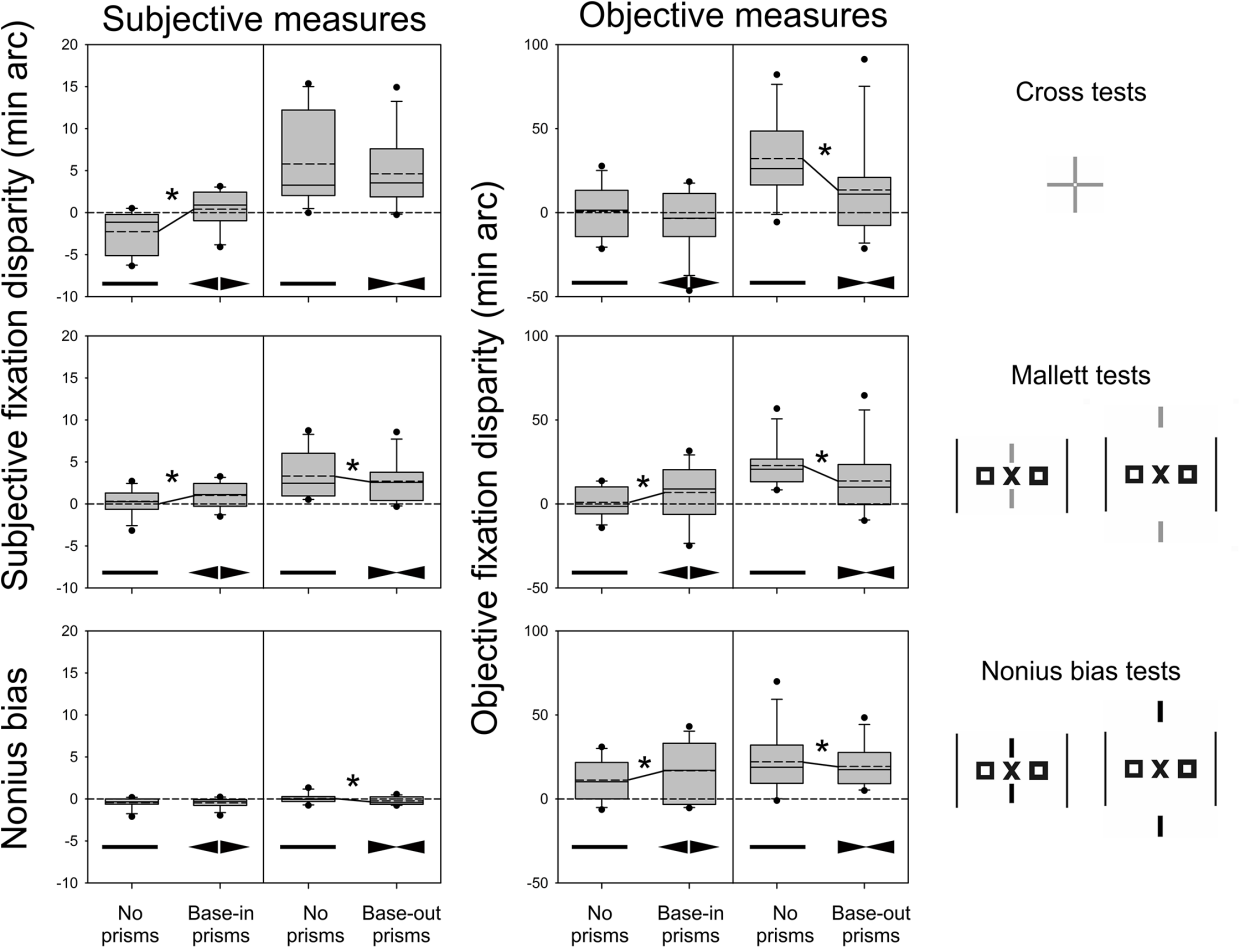
Subjective and objective measures for the three types of tests measured with and without prisms. Box-plots are presented separately for base-out and base-in prisms. Positive and negative values mean an over-convergence (eso) or under-convergence (exo), respectively. In each box, the line indicates the median and the dotted line the mean. Significant mean prism effects are indicated by an asterisk and a line that connects the two mean values. Each box represents 12 participants.

Table 2 gives an overview of the complete statistical results for the subjective and objective measures, i.e. the parameters of the linear mixed effects models, for each of the three types of tests (Cross test, Mallett tests, Nonius bias tests) and for the two groups receiving base-in or base-out prisms. For illustration, Fig 6 shows the corresponding box-plots. Note that the subjective Nonius bias test does not give a fixation disparity, but a measure of the nonius offset required for perceived alignment of binocularly presented nonius lines; the Nonius bias tests provide a fixation disparity only with objective eye movement recordings. Therefore, in Table 2 and Fig 6 from the 12 comparisons between the prism and the no prism condition, 10 comparisons refer to a fixation disparity. In eight of these ten conditions, significant effects of wearing prisms were found and all eight went in the expected directions: thus, base-in prisms produced a shift in the eso (positive) direction and base-out prisms in the exo (negative) direction. The amount of the Cohen effect sizes ranged between 0.22 and 1.29 (median 0.62); five of these eight effect sizes were above 0.5 which therefore are considered as medium or large effects.

### Objective and subjective fixation disparity: correlations and prism effects

**Fig 7:**
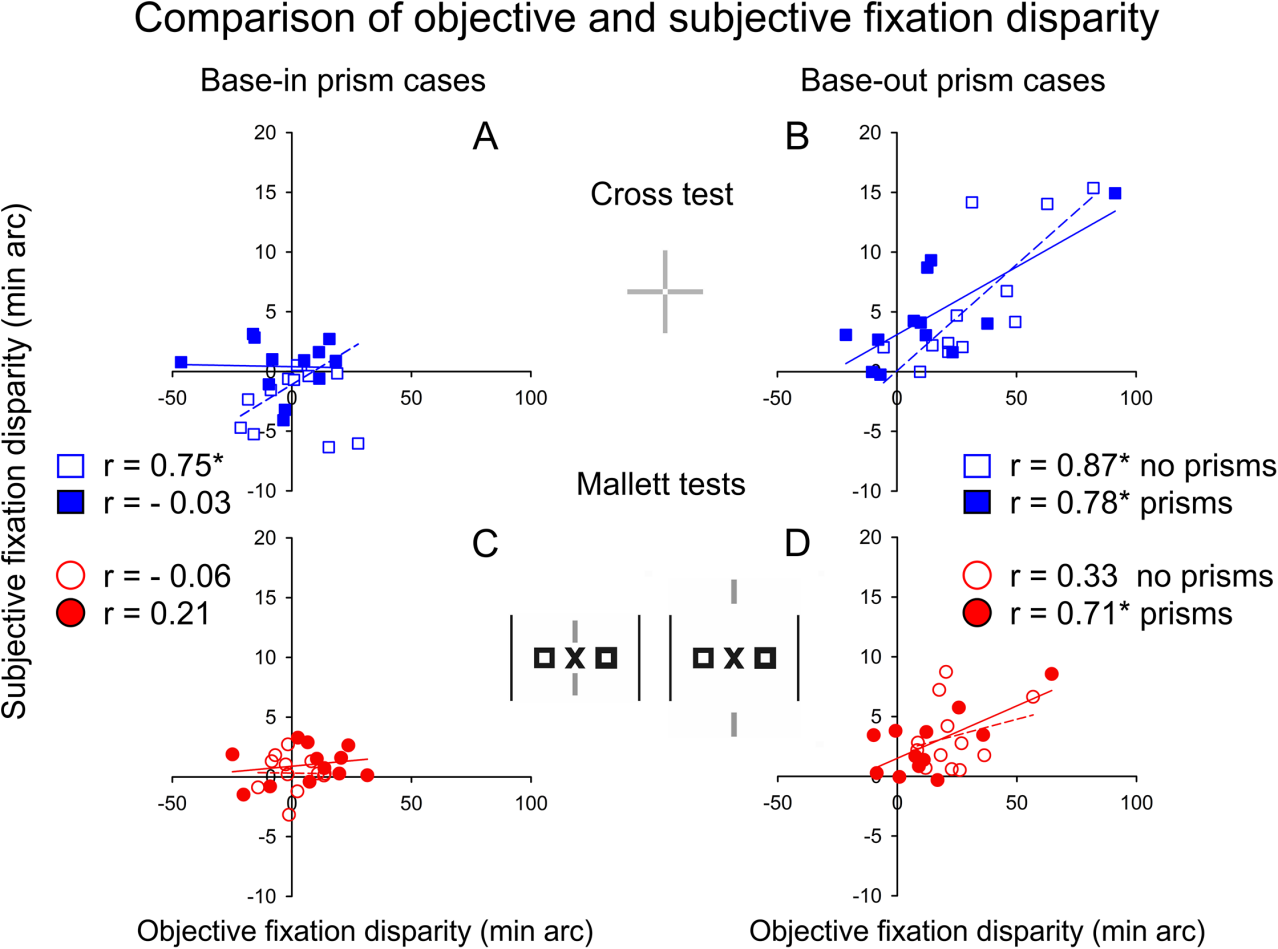
Relation between subjective and objective fixation disparity. Robust regression analyses between these two measures in the two conditions with and without prisms, separately for the Cross test and the Mallett tests and participants who received base-in or base-out prisms (n = 12, respectively). Positive and negative values mean an over-convergence (eso) or under-convergence (exo), respectively. These robust correlations were significant as indicated by asterisks with one-tailed p < 0.005 if r > 0.70.

Fig 7 illustrates correlations between objective and subjective fixation disparity (oFD and sFD) for the Cross test and the Mallett tests, both in the condition without and with prisms. The objective fixation disparity is used as independent variable, for comparison with the slope in a previous study [33]. Four of these eight correlations were above 0.70 which is significant (p < 0.01, one-tailed). There is a tendency that this correlation is higher with the Cross test and among participants receiving base-out prisms. Note that the amount of fixation disparity is much larger for the objective than for the subjective measure.

Further analyses refer to correlations between prism effects in objective versus subjective fixation disparity. To facilitate comparisons of prism effects in the two types of fixation disparity with considerably different amounts, the individual changes (in the raw unit of minutes of arc) were normalized by the pooled standard deviation of each group (base-in, base-out) and each test (Cross test, Mallett tests). This leads to individual effect sizes d_i_

$$d{}_{i}(FD)=\frac{\left(FD_{prism}-FD_{noprism}\right)}{\sqrt{(({SD}_{prism}^{2}+{SD}_{noprism}^{2}})/2)}$$

**Fig 8:**
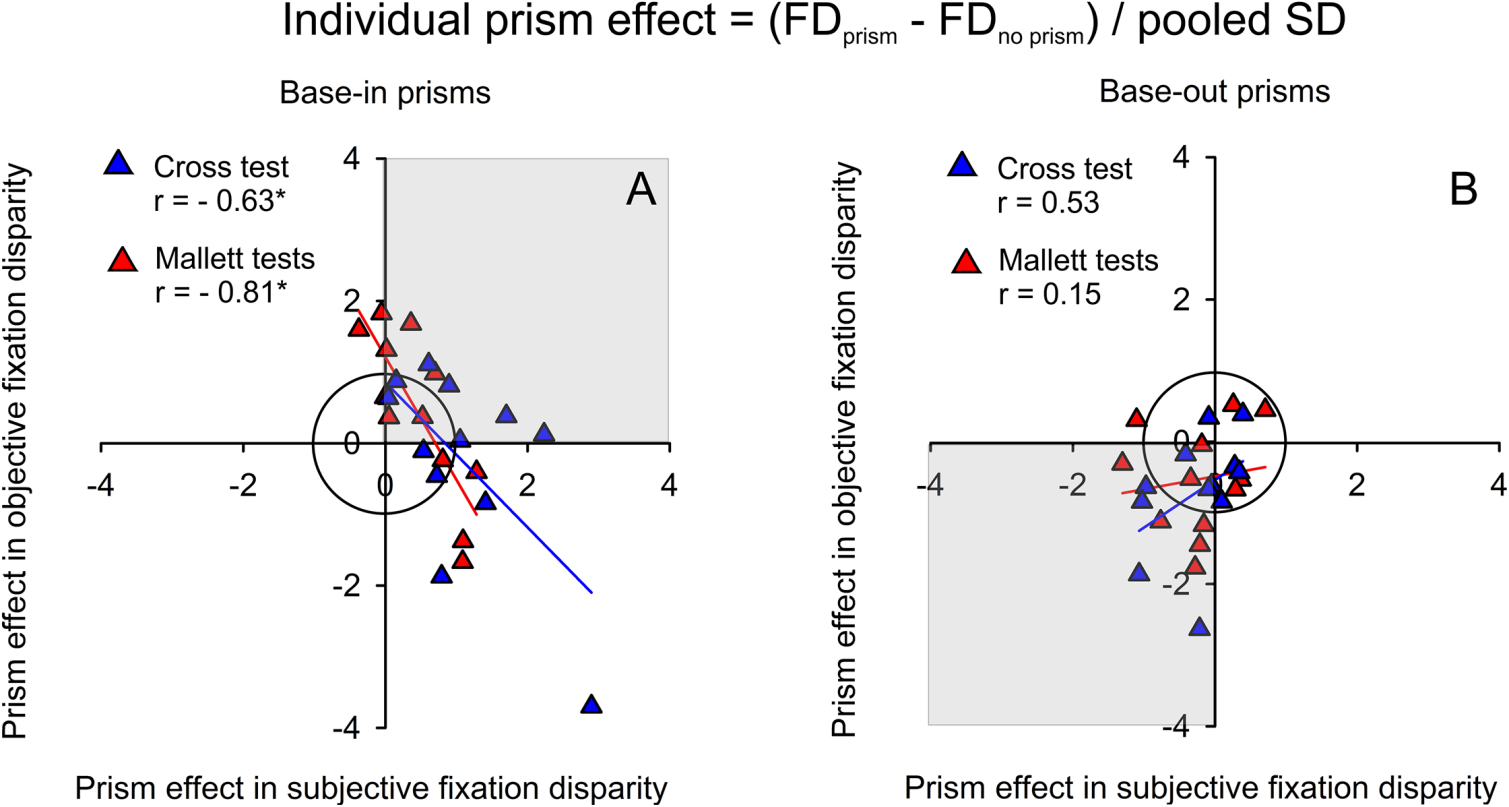
Relation between individual prism effects sizes of subjective versus objective fixation disparity, i.e d_i_(sFD) versus d_i_(oFD). Data are presented separately for base-in and base-out prisms (n = 12 participants, respectively) and for the Cross test and the Mallett tests. The grey quadrants indicate where individual data points are expected based on the hypotheses, that shifts should be in the positive (eso) direction with base-in prisms and in the negative (exo) direction with base-out prisms. The inner circle has a radius of 1.0 and indicates that individual data points near or outside the circle are statistically large. Regression lines, correlation coefficients and p-values (p < 0.05, two- tailed, indicated by asterisks) are based on robust regression analyses.

Fig 8 shows the correlation between the d_i_-values of the two types of fixation disparity, separately for the Cross test and the Mallett tests. The subjective individual effect sizes were used as independent variables since the prism prescription is based on subjective tests (see Fig 3) and objective fixation disparity may change as dependent variable. The group means of the individual effect sizes d_i_ are included in Table 2 as Cohen’s d-values. The circle included in Fig 8 has a radius of 1.0; thus, data points near and outside this circle represent statistically large effects in these observers, while data points close to zero show negligible or no effects. This graph has the advantage to show the amount of effect sizes in objective and subjective fixation disparity in relation to each other and using a comparable, same quantitative scale. The grey quadrants in these graphs indicate the hypotheses that an exo shift is expected for base-out prisms and an eso shift for base-in prisms. For base-out prisms (Fig 8B), 9of 10 data points with large effect sizes (outside the circle) are clearly within the expected grey quadrant, which reflects the mean effects reported in Table 2; the correlations between d_i_(sFD) and d_i_(oFD) of 0.53 and 0.15 were insignificant, however (p = 0.077 and 0.651, two-tailed). More surprising are the following results for base-in prisms. Many cases with large effect sizes were not in the expected grey quadrant and significant negative correlations were found, suggesting that d_i_(oFD) decreases as d_i_(sFD) increases. Moreover, d_i_(oFD) becomes even negative when d_i_(sFD) exceeds a level of 1.0. This pattern of individual results was unexpected, but is confirmed by the replication of significant negative correlations for the Cross test (r = - 0.63, p = 0.027, two-tailed) and the Mallett tests (r = - 0.81, p = 0.001).

### Effects of fusional vergence reserves

One could hypothesize that prism effects are smaller in those participants with a more adaptive vergence system, i.e. in those with a larger fusional vergence reserve in the direction of the prism. Further, this fusional vergence reserve may be correlated with the amount of prism, since—at a certain associated phoria—larger fusional vergence reserves lead to more flat fixation disparity curves and larger aligning prisms. Accordingly, we found that the amount of prism was positively correlated with the fusional vergence reserve in the same direction, both for base-in prisms (r = 0.56, p = 0.031, one-tailed) and base-out prisms (r = 0.65, p = 0.011). However, the fusional vergence reserve in the opposite direction was not correlated with the amount of prism, neither for base-out prisms (r = 0.17, n. s.) nor for base-in prisms (r = 0.04, n. s.).

**Fig 9:**
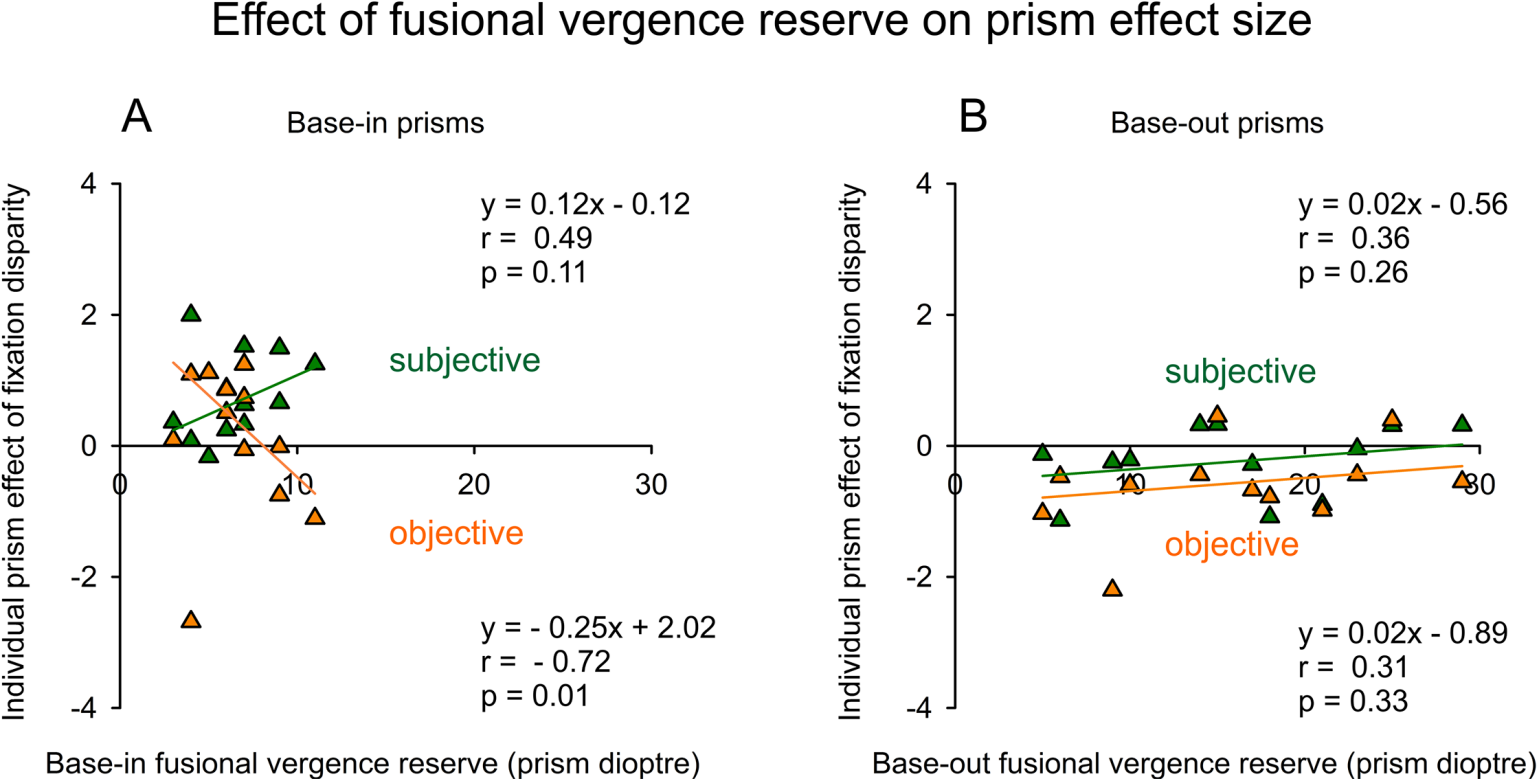
Effect of the fusional vergence reserve on the individual prisms effect sizes. Regression analyses are presented for subjective [d_i_(sFD)] and objective [d_i_(oFD)] prism effect size, separately for base-in and base-out prisms. The data of the Cross test and the Mallett tests were combined. The regression equations, correlation coefficients and two-tailed p-values are based on robust statistical procedures (n = 12).

In Fig 9, the individual prism effect sizes are plotted as a function of the fusional vergence reserve; data of the Cross test and the Mallett tests were combined because of their similar results in Fig 8. For base-out prisms (Fig 9B), the regression lines suggest that the prism effects (in the hypothesized negative direction) tended to become smaller (more positive) when the base-out fusional vergence reserve increased (Fig 9B). The slope was similar for objective and subjective fixation disparity (0.02), but the correlations of 0.31 (p = 0.327, two-tailed) and 0.36 (p = 0.257) were not significant. Base-in prisms showed different results for the two types of fixation disparity (Fig 9A). For objective fixation disparity, the hypothesized positive prism effect significantly declined (r = - 0.72, p = 0.012, two-tailed) as the fusional vergence reserve increased as it was expected since larger reserves indicate a more adaptive vergence system and, thus, prism effects may be smaller. Note that one outlier in the objective prism effect size had virtually no effect on the regression line due to the robust regression that was applied to all 12 data points. For subjective fixation disparity, an opposite relation was found: against the expectation, the prism effect significantly increased as the fusional vergence reserve increased. However, this effect did not reach significance (r = 0.49, p = 0.109, two-tailed). The two crossing regression lines suggest an interaction which can be described by the difference between the objective and subjective effect sizes for base-in prisms, i. e. d_i_(oFD)–d_i_ (sFD): this difference was significantly correlated with the fusional vergence reserve (r = - 0.76, p = 0.0065, two-tailed), as shown by a robust linear regression. For base-out prisms, this difference apparently did not depend on the fusional vergence reserve, as shown by the parallel regression lines in Fig 9B.

## Discussion

**Fig 10:**
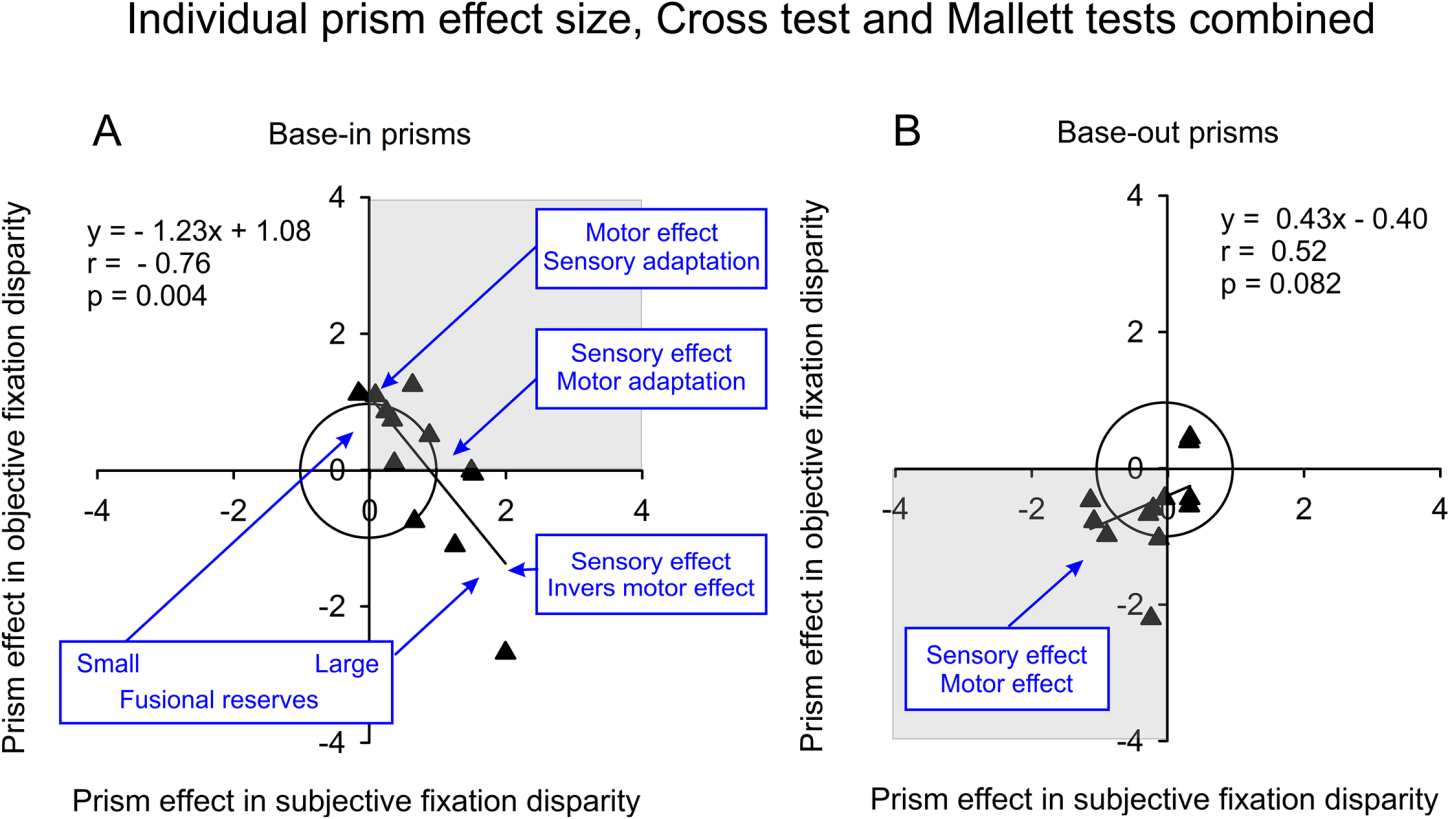
Interpretation of the relation between individual prism effects sizes of subjective versus objective fixation disparity, i.e. d_i_(sFD) versus d_i_(oFD). The combined data of the Cross test and the Mallett tests are presented separately for base-in and base-out prisms. The grey quadrants indicate where individual data points are expected based on the hypotheses, that shifts should be in the positive (eso) direction with base-in prisms and in the negative (exo) direction with base-out prisms. The inner circle has a radius of 1.0 and indicates that individual data points near or outside the circle are statistically large. The regression equations, correlation coefficients and two-tailed p-values are based on robust statistical procedures (n = 12). The blue insets describe the working hypothesis of the underlying physiological mechanisms (see Discussion).

In this study, participants wore eyeglasses with prisms over about 5 weeks in everyday life in order to improve binocular vision, i.e. to correct for a fixation disparity. Two extreme potential outcomes could have been hypothesized beforehand: (1) the fixation disparity might have been reduced to zero if the prisms had the full effect or (2) the fixation disparity might have remained the same if vergence adaptation had diminished all prism effects. The present study showed an intermediate pattern of results in that objective and subjective fixation disparity changed in the expected directions, at least in most participants. However, some specific effects occurred depending on the type of fixation disparity (objective versus subjective) and the direction of the prisms (base-in versus base-out). The interpretation of these effects is facilitated by Cohen’s effect sizes for group mean effects (d) and individual effects (d_i_). Both of these types of prism effect sizes were calculated relative to the pooled standard deviation in the actual group and condition. In this way, effects of the prisms could quantitatively be compared despite the fact that the prism effect (in the raw unit of minutes of arc) was much larger in objective than in subjective fixation disparity. Scatter plots of the individual prism effect sizes for subjective versus objective fixation disparity describe the prism effects in a sensory-oculomotor domain. For the following discussion, Fig 10 illustrates the averaged findings of the Cross test and the Mallett-tests, that gave similar results (see Fig 8).

### Subgroup wearing base-out prisms

The subgroup of 12 participants with base-out prisms (those who initially had an eso associated phoria at 6 m distance) showed a pattern of results that might have been expected. First, the initial objective and subjective fixation disparity (measured at 6 m distance) had an eso direction in most cases and the two measures were correlated with r-values in the order of 0.7 (Fig 8). Smaller correlations of 0.23 and 0.49 were found in a previous study [33] where a 60 cm viewing distance was used and the majority of participants had an exo fixation disparity.

The present study found that wearing base-out prisms induced mean shifts of subjective and objective fixation disparity in the expected exo direction in several conditions (Fig 6). The mean prism effect size tended to be larger for objective fixation disparity (Cohen’s d = - 0.69,- 0.53,- 0.17 for the three tests) than for subjective fixation disparity (d = - 0.23,- 0.23). On the individual level (Fig 10B), objective prism effect sizes were in the range from—2 to 0.5 and tended to be positively correlated (r = 0.52, p = 0.08, two-tailed) between the objective and subjective fixation disparity. All 5 cases with large effect sizes (d_i_(sFD) > 1, d_i_(oFD) > 1, outside the circle) were negative, as expected from the hypotheses. Both prism effects tended to decline as the fusional vergence reserve increased (r = 0.31 and r = 0.36 in Fig 9), i. e. as the adaptability of the vergence system increased. This trend was expected, but was insignificant in the sample of 12 participants.

Thus, objective and subjective fixation disparity showed similar properties since they were positively correlated (without and with prisms) and responded in a similar way to wearing prisms. Base-out prisms induce a forced convergence, i.e. prisms in the present study (up to 8 Δ, for both eyes together) induced an increase of convergence up to 4.6 deg. This corresponds to a change in viewing distance from 6 m to about 70 cm. Such changes occur in everyday vision and were shown to produce exo shifts by about 2 min arc in subjective fixation disparity [44] and by about 5 min arc in objective fixation disparity [13], on the average. In comparison, the mean shifts due to wearing base-out prisms in the present study were 0.34 min arc for subjective fixation disparity and 10 min arc for objective fixation disparity, when a central fusion stimulus was present as in the Mallett tests. The situation was very much different in the other group wearing base-in prisms.

### Subgroup wearing base-in prisms

Base-in prisms induced significant shifts in mean fixation disparity in the expected eso direction (except for objective fixation disparity at the Cross test). However, the results can only fully be described by correlations between individual objective and subjective prism effect sizes that–surprisingly–showed a significant negative correlation of r = - 0.76 (p = 0.004, two-tailed): the larger the observer’s effect in subjective fixation disparity, the smaller was the effect in objective fixation disparity (Fig 10A). This negative correlation would also appear based on the raw effects in minutes of arc, i.e. without the normalization by the pooled group standard deviation to calculate the effects sizes. However, the effect sizes have the advantage to allow for the following interpretation of subjective and objective effects on the same scale where effects sizes larger than 1 are considered as large. Three particular conditions can be identified along the regression line with the following interpretation: (1) d_i_(sFD) = 0 and d_i_(oFD) = 1, meaning a sensory adaptation (no change in the perception of dichoptically presented nonius lines, i.e. no change in subjective fixation disparity) and a motor effect (change in the oculomotor vergence error, i.e. a change in objective fixation disparity), (2) d_i_(sFD) = 1 and d_i_(oFD) = 0, meaning a sensory effect and motor adaptation, and (3) d_i_(sFD) > 1 and d_i_(oFD) < 0, meaning a large positive sensory effect as expected and a large negative motor effect opposite to the expected direction. This negative regression between objective and subjective effects does not correspond to the conventional prior hypotheses, but was replicated within this study with the Cross test and the Mallett tests (Fig 8A and Fig 10A), where individual objective prism effects sizes ranged from + 1.8 to – 3.8 for the Cross test while the group mean effect size was only - 0.25. Thus, the mean result does not reflect the underlying pattern of result.

A further interpretation is based on the fusional vergence reserves and the amount of base-in prisms. These two optometric parameters were significantly correlated as expected (r = 0.56): subjects with a more adaptive vergence system have a larger fusional vergence reserve and require larger prisms. The robust regression lines in Fig 9 were almost unaffected by outliers and allow for the following interpretation. Subjects with a small base-in fusional vergence reserve, i.e. those who are not able to diverge and who tended to receive small prisms, showed no subjective (sensory) effect, but a marked eso objective (motor) effect. The more the fusional vergence reserve increased, the more the objective effect declined and the subjective effect tended to increase. In subjects with an average base-in fusional vergence reserve of about 6 Δ, both the objective and the subjective prism effect was about 0.6 and in the expected positive (eso) direction. Finally, participants with a larger fusional reserve, i.e. those who are able to strongly diverge and tend to receive larger prisms, showed no objective (motor) effect, but a marked eso subjective (sensory) effect. This interpretation is also supported by the following result of a linear mixed-effects regression (Table 2) which includes the interaction term “Prism effect: Amount of prism”: significant positive coefficients of 5.69 and 3.97 were found for the objective fixation disparity with the Mallett tests and with the Nonius bias tests, suggesting that these prism effects were larger with smaller amounts of prisms; note that base-in prisms are coded with a negative sign in these calculations (Table 1).

The negative slope of the regression in Fig 8A and Fig 10A is mainly the result of three participants with base-in prisms who had substantial negative objective effect sizes with amounts larger than 1, which were replicated in both the Cross tests and the Mallett tests. The most negative objective effect size of - 2.7 in Fig 10A fits well within the regression of the remaining sample, while this participant was a clear outlier in Fig 9 with respect to the fusional vergence reserves. These three participants with large objective effect sizes in the lower right quadrant wore base-in prisms of - 3,- 3.5, and - 4.5 Δ and had unexpected negative (exo) objective effects while the subjective effects were positive (eso) as predicted. Interestingly, this unexpected result is compatible with findings in the following two studies. Simonsz and Bour [15] applied base-in prisms of similar amount to three subjects (- 3, - 4, and - 5 Δ): they found that all three observers showed an expected eso shift in subjective fixation disparity, while two observers showed a surprising exo shift in objective fixation disparity; this finding resembles the negative (exo) objective effect in the present study. Brautaset and Jennings [32] applied smaller base-in prisms of - 1.86 Δ and found changes of objective and subjective fixation disparity in the expected eso (positive) direction in 5 observers. Since the prisms were larger in the Simonsz and Bour [15] study and smaller in the Brautaset and Jennings study [32], one may expect larger and smaller fusional vergence reserves in these two studies so that the opposite (negative vs. positive) objective prism effects in the latter two earlier studies seem to be compatible with the scheme in Fig 10 that resulted from the present study (that included larger and smaller prisms). Note that these three studies used long viewing distances: 6 m in the present study, 3.4 m in Simonsz and Bour [15] and 4 m in Brautaset and Jennings [32]. For comparison, Kertesz and Lee [12] tested 4 observers at 51 cm and generally found effects in the expected direction in both types of fixation disparity in response to convergent and divergent 4 deg disparities that correspond to base-out and base-in prisms, respectively. This review of the limited data base in the literature of short-term forced vergence load suggests that not in all cases the objective fixation disparity changes in the direction as expected by the typically negative slope of fixation disparity curves. Depending on conditions and observers, positive slopes of fixation disparity curves may occur.

However, the scheme of interpretation in Fig 10 was not expected beforehand and the data base is rather small with only 12 participants in each group. Since research in this field is very limited, this scheme is suggested as a working hypothesis that needs further investigation. Moreover, given the marked individual differences in binocular vision, this scheme may apply to many, but not to all subjects (see also the outlier in Fig 9).

### Dichotomy of base-out/base-in prisms and subjective/objective fixation disparity

The present response patterns were influenced by two types of dichotomic physiological dimensions: the different effects of wearing base-in or base-out prisms and also the different results for subjective and objective fixation disparity (at least for base-in prisms).

First, the two prism directions introduce different conditions of forced vergence. Base-out prisms induce a ocular convergence load that resembles the convergence that is exerted naturally in all near vision tasks. The convergence near point is typically less than 10 cm, showing that the eyes are able to strongly converge the visual axis. In contrast, base-in prisms introduce a divergent state that the eyes never exert in natural vision. With the present base-in prisms up to 5 Δ, this state of divergence is up to about 3 degree. The vergence system has to adapt to this non-natural state in order to shift the initial exo associated phoria in these observers (measured with subjective tests) into a more eso state. A consequence of this difference in the two prism directions is the conventional result of testing fusional vergence reserves: convergence break points are typically much larger than divergence break points. Accordingly, when testing forced-vergence fixation disparity curves, the slope is typically more flat in the convergent (base-out) than in the divergent (base-in) direction. Thus, particularly large base-in prisms shift the vergence system in a non-natural condition, which may be necessary–however–to reduce the exo fixation disparity. Jenkins [57] found an improvement in distance vision binocular acuity that was more pronounced with base-in prisms than with base-out prisms.

Second, the two types of fixation disparity are not at all equivalent physiological properties that are measured with different techniques (nonius lines versus eye trackers). The different nature was described in the Introduction. Objective fixation disparity refers to the oculomotor vergence error relative to the assumed optimal vergence state when the binocular target is projected onto the centre of the foveola (corresponding to the monocular eye positions during calibration) and subjective fixation disparity reflects an incomplete sensory/neural transfer of the visual direction of the fused binocular target to the monocular nonius lines. Given these different physiological mechanisms, these two measures may not react in the same way. E. g. adaptation may operate in a different way in the sensory domain versus the oculomotor domain. Such differences were found in two aspects: first, the shift to a more divergent state over the one minute recording period occurred consistently only in objective, but not in subjective fixation disparity; see Supporting Information (S2 Text); second, the prism effects were different in the two types of fixation disparity for base-out versus base-in prisms. When wearing base-out prisms (in the more natural mode of converging the eyes), the subjective and objective fixation disparity seems to respond in a similar, concurrent way. However, when wearing base-in prisms, the response pattern becomes more complicated and depends on individual factors. Observers with smaller fusional vergence reserves (who tend to receive smaller prisms inducing less divergent states) showed a concurrent shift of both types of fixation disparity in the expected eso direction. Observers with larger fusional vergence reserves (who tend to receive larger prisms inducing more divergent states) showed the expected eso shift in subjective fixation disparity, but a shift of objective fixation disparity in the opposite, i.e. exo direction. It seems as if the absolute divergent state with large base-in prisms may be the particular condition when an opposite objective response pattern occurred. This a-posteriori interpretation is suggested as a working hypothesis that requires further investigation.

### The size of the prism effects: aspects of statistics, physiology, and practical relevance

The angular amounts of these effects were in the order of minutes of arc, so that–although being statistically significant—the question arises whether these prism effects are large enough to be physiologically meaningful and practically relevant. This is discussed in two steps.

First, effect sizes have been reported, i.e. changes in minutes of arc relative to the pooled standard deviation of a variable in the corresponding group and condition. For group mean values, the majority of these Cohen effect sizes d were in the range of 0.5 to 1.0 which are conventionally interpreted as medium to large effects. Thus, mean effects reached up to about one standard deviation of the present sample. In the base-in prism group, the average effect was less relevant, rather the individual effect sizes showed correlations between subjective and objective fixation disparity and these were similar or larger than 1.0 in the majority of the base-in cases. Thus, when measured relative to the distribution in the sample, the present prisms effects can be interpreted as substantial.

Second, the practical and clinical relevance of the amount of the effects in fixation disparity can be estimated in relation to effects in other studies (when comparable conditions with a central fusion stimulus were used). The present 0.5 min arc eso shift in subjective fixation disparity with the Mallett tests resembles the 0.6 min arc eso shift in subjective fixation disparity that was found when gaze direction was lowered from horizontal to 25 deg downwards [92]. The latter effect may be ecologically advantageous for near vision. Comparisons between groups showed that subjects with visual symptoms had a 0.5–1.0 min arc more exo subjective fixation disparity (in near vision) compared to non-symptomatic subjects [49, 50, 98]. With respect to objective fixation disparity, the mean prism effect with a central fusion stimulus was about 5–10 min arc. For comparison, the objectively measured vergence angle during an experimental reading task shifted by 6.5 min arc in the exo direction when sentences were blurred [99]. Further, some studies provided interindividual correlations between objective fixation disparity and heterophoria which is a clinically applied measure. Observers who differ in objective fixation disparity by 10 min arc (corresponding to the present effect with the Mallett unit), differ in heterophoria by about 1.5 deg [20, 33]. These comparisons in terms of minutes of arc suggest that the amount of these changes in fixation disparity due to wearing prisms were in a range that can be physiologically relevant for the quality of vision.

### Limitations, potentials and perspectives of the study

Video eye tracking has the advantage to allow convenient recordings, but may be susceptible to pupil artifacts: changes in pupil size may shift the centre of the pupil and may therefore affect the detected eye position [93, 94]. Our quantitative estimations of the pupil effects (S2 Text) suggest that this potential artifact cannot explain the reported prisms effects in objective fixation disparity. Future research in this field should carefully control the pupil size to estimate potential effects.

In this study, observers with binocular imbalance were not included; this would have introduced a larger variance in all measures. However, these clinically more relevant subjects are candidates to benefit from wearing prisms. Particularly, prism effects may be stronger in clinical cases with a non-adaptive vergence system. This was already suggested by the influence of the fusional vergence reserves in the present study.

The practitioner–using subjective tests–may be interested in effects that can be replicated within a single client with clinical test methods. However, the present results are based on group means (n = 12) and correlations within the groups, that have been measured with many repeated tests within each observer. The reported effects may be difficult to find with faster and less precise clinical test procedures for subjective fixation disparity. Therefore, practitioners may have come to the experience that wearing prisms has no measurable effects in fixation disparity. Objective recordings have not been made clinically. The present results suggest, however, that with an extension and improvement of test procedures, objective effects of prism wear may be detected.

Despite the observed prism effects in both types of fixation disparity, we cannot conclude on the clinically important question whether prisms should be worn or not. For this question one has to consider whether visual complaints are reduced and visual functions are improved by the prisms. Lie et al. [72, 73] had prescribed prisms following the MCH procedure in a sample of asthenopic subjects and found improved relative accommodation and vergence measures (i.e. fusional vergence reserve) directly after the prescription, one and even five years later. Moreover, in four participants who discontinued wearing the prisms, visual functions deteriorated again.

The present sample size was large enough to show prism effects in this type of an experimental study, however, the sample was not sufficient to provide representative results. Therefore one should be careful regarding the direct transfer of the present results to clinical applications, also because of individual differences in the vergence and fusion system that lead to individually different prism effects (as shown in this study). Thus, more extended studies are necessary and could include a control group that was not used here.

The present findings refer to tests at a 6 m viewing distance which is the distance where prisms are prescribed by the “Measuring and Correcting Methodology after H.-J. Haase“. However, the results are not specific for this method. Rather, the study more generally reports on whether wearing base-in or base-out prisms leads to sustained changes in fixation disparity in the hypothesized directions. The present procedure aims to correct the vergence resting position, where near vision is not involved. The classic Mallett unit [2, 48] is predominantly used in near vision at 40 cm since the resulting near aligning prism in near vision has been shown to be related to visual symptoms [49, 51, 52, 99]. Therefore, future research should also include near vision tests of objective and subjective fixation disparity.

As a perspective, this study proposes a methodology for the simultaneous recording of subjective and objective fixation disparity. These parameters may—in future research—be investigated in several conditions: (a) forced-vergence fixation disparity curves while vergence adaptation is eliminated, (b) adaptation to sustained fixation and to prisms as a function of exposure duration, and (c) after long-term adaptation during wearing prism eyeglasses.

### Conclusions

In this study of wearing prisms over about 5 weeks, objective and subjective fixation disparity were correlated¸ but the type of the fusion stimulus and the direction of the required prism may play a role. Fixation disparity was not reduced to zero by the prisms (probably due to vergence adaptation), but significant changes in fixation disparity with large effect sizes were observed. Participants receiving base-out prisms showed hypothesized effects, which were concurrent in both types of fixation disparity. In participants receiving base-in prisms, the individual effects in subjective and objective fixation disparity were negatively correlated: the larger the subjective (sensory) effect, the smaller the objective (motor) effect. This response pattern was related to the vergence adaptability, i.e. the individual fusional vergence reserves. These findings are suggested as working hypothesis for further research of the relation between sensory and motor effects of wearing prisms.

## Supporting Information

S1 Text Nonius bias test. (DOCX)

S2 Text Effects of pupil size. (DOCX)
