# Supplementary material for: Effects of Prism Eyeglasses on Objective and Subjective Fixation Disparity
Source: PLoS One. 2015 Oct 2;10(10):e0138871. doi: 10.1371/journal.pone.0138871 (PMC4592239; doi:10.1371/journal.pone.0138871)
Supplement: S1 Text — (DOCX) [file pone.0138871.s001.docx]

**Supporting Information S1: Nonius bias test**

The nonius bias refers to the offset required for perceived alignment of binocularly presented nonius lines; this effect can be understood as a slight spatial distortion of visual directions in binocular vision, when the nonius lines have a vertical separation from a central target [84, 85]. The present data set allows investigating properties of the nonius bias regarding two questions.

(1) It was reported that the nonius bias is correlated with subjective fixation disparity [84, 85]. This was confirmed in the present study in the condition without prisms (r = 0.62, p < 0.001, one-tailed) and a trend occurred in the condition with prisms (r = 0.33, p = 0.061); all 24 participants were combined for this analysis, irrespective whether they had an eso or exo associated phoria (as in a previous study [85]). This correlation may suggest that wearing prisms may not only affect the fixation disparity, but also the nonius bias. In fact, although the subjective nonius bias is not a vergence measure, it was significantly changed by wearing base-out prisms in the negative direction by - 0.29 min arc (d = 0.55); see Fig. 6 and Table. 2. The direction of this effect corresponds to the expected negative (more exo) condition in subjective fixation disparity. The base-in group, however, had no significant effect in the subjective nonius bias. These findings suggest that the nonius bias was modified, when an eso fixation disparity was changed by wearing base-out prisms over 5 weeks. However, short exposures of prisms during experimental sessions did not change the nonius bias [100]. Clinical experience with MCH-prism correction suggests that observes with large associated phoria can have distorted space perception that can be improved by wearing prisms [67, 101].

(2) Gerling et al. [69] made an experiment with a modified Pointer test (Fig. 3) where they switched from a fully binocular presentation of pointer and scale to the conventional dichoptic presentation: they found that the objectively recorded vergence position changed to a more eso condition by 7 min arc, on the average, in a sample of subjects that mainly received base-out prisms following the MCH-procedure. Thus, the objective vergence position may be influenced by the dichoptic nature of nonius targets that are required for testing subjective fixation disparity. This can be investigated with the present data by comparing the objective fixation disparity tested with the Nonius bias tests (binocular nonius lines) versus the Mallett tests (dichoptic nonius lines). The box-plots in Fig. 6 illustrate the distributions in these measures and Table S1 shows the statistics of a comparison. Regression analyses between objective fixation disparity tested with the Nonius bias tests (oFD_Non_) versus the Mallett tests (oFD_Mal_) showed highly significant correlations suggesting that both measures reflect the inter-individual variance. For subjects receiving base-in prisms, the Nonius bias tests gave a significantly (p = 0.002) more eso objective fixation disparity than the Mallett tests. This finding tends to correspond to the one of Gerling et al. [69] and suggests that fixation disparity may be affected by the dichoptic nature of nonius lines (which are required for subjective testing). This effect was correlated with the subjective fixation disparity at the Pointer test in the study of Gerling et al. [69], while no relation to the subjective fixation disparity was found in the present study.

These findings provide tentative evidence that the dichoptic nature of nonius lines may affect the objective fixation disparity. The underlying mechanism remains to be investigated in future research. Provisionally, we conclude that dichoptic nonius lines may not be included in tests for measuring the natural vergence state. But dichoptic nonius lines are required for measuring the subjective fixation disparity (in relation to objective fixation disparity, as in the present study). Note, that significant prism effects in objective fixation disparity were also found when no dichoptic targets were present, i.e. with the Nonius bias tests. (Table 2).

References

100. Jaschinski W. Fixation disparity and accommodation as a function of viewing distance and prism load. Ophthalmic Physiol Opt. 1997;17: 324-339.

101. Brückner R. The correction of heterophorias with fixation disparity. Optometrie. 1989;1: 27-42.

**Table S1.** Comparison of objective fixation disparity tested with the Nonius bias tests (oFD_Non_ ) versus the Mallett tests (oFD_Mal_). Regression analyses and comparisons of mean values are shown separately for participants receiving base-out and base-in prisms, and the test condition without and with prisms. Robust statistical procedures are applied.

|  |  | **Regression analysis** | | | **Comparison of mean objective fixation disparity: Mallett tests versus Nonius bias tests** | |
| --- | --- | --- | --- | --- | --- | --- |
|  |  | **Regression line** | **Correlation coefficient** | **p-value (one-tailed)** | **Mean difference (min arc)** | **p-value (two-tailed)** |
| **Base-in cases** | **No prisms** | oFD_Non_ = 0.96 oFD_Mal_ + 9.43 | r = 0.75 | p = 0.0025 | 9.25 | 0.002 |
|  | **Prisms** | oFD_Non_ = 0.90 oFD_Mal_ + 10.70 | r = 0.89 | p = 0.0001 | 10.04 | 0.002 |
| **Base-out cases** | **No prisms** | oFD_Non_ = 1.01 oFD_Mal_ - 1.02 | r = 0.71 | p = 0.0045 | -0.68 | 0.869 |
|  | **Prisms** | oFD_Non_ = 0.57 oFD_Mal_ + 11.56 | r = 0.92 | p = 0.0001 | 5.96 | 0.0660 |
