# Supplementary material for: Effects of Prism Eyeglasses on Objective and Subjective Fixation Disparity
Source: PLoS One. 2015 Oct 2;10(10):e0138871. doi: 10.1371/journal.pone.0138871 (PMC4592239; doi:10.1371/journal.pone.0138871)
Supplement: S2 Text — (DOCX) [file pone.0138871.s002.docx]

**Supporting Information S2: Effects of pupil size**

In video eye tracking, variations in pupil size may affect the measures of objective fixation disparity since a shrinking of the pupil can shift the centre of the pupil to a more nasal position which may appear as an artificially more eso vergence position; a pupil dilatation has the opposite effects, respectively. For a quantitative account of this potential methodological artifact, two steps of analyses were made: (1) effects of time during the 60 second recording period and (2) effects of wearing the prisms.

(1) Over the 60 second recording period, the estimated pupil became smaller which could best be approximated by a ln-transformation of time(s). For the two groups and three type of tests, the time coefficients ranged between - 0.12 and - 0.15 mm/ln(time(s)/1s) with an average slope of - 0.13 mm/ln(time(s)/1s); this means a shrinking of about 0.54 mm/minute. A 0.54 mm smaller pupil would artifactually lead to a more eso objective fixation disparity by 16 min arc (= 0.54 * 30 = 16 min arc, see Methods [93, 94]). In contrary, a time shift of objective fixation disparity in the opposite direction was observed: all tests and prism conditions showed divergent time shifts with slopes between - 6.7 and - 10.52 min arc/ln(time(s)/1s) (Table 2). The mean slope was - 8.07 min arc/ln(time(s)/1s), which means an exo shift of about 33 min arc within the one minute recording period. This divergent time trend cannot be explained by the pupillary time trend since the potential pupillary artifact would produce a convergent trend. If any, the divergent trend could only be an underestimation of the true objective divergent trend due to the potential pupillary artefact. Moreover, the 6 coefficients of the oFD time trend (ranging from – 6.7 to – 10.5 min arc/ln(time(s)/1s)) were not correlated (r = 0.18, n = 6) with the coefficients of the pupillary time trend (ranging from - 0.12 to ‑ 0.15 min arc/ln(time(s)/1s)). Thus, the divergent trend in objective fixation disparity in the course of the 60 second recording period cannot be explained by the concurrent shrinking of the pupil.

Subjective fixation disparity showed significant time effects in the convergent direction in the base-out group, both for the Cross test (0.72 min arc/ln(time(s)/1s); p < 0.001) and for the Mallett tests (0.18 min arc/ln(time(s)/1s); p = 0.027). However the base-in group did not show significant time effects. Thus, the time effect can have different directions in objective and in subjective fixation disparity, at least in the base-out group.

(2) The prism effect is the main experimental factor in this study, so it is important to consider potential artifacts due to the pupil size. For the three tests and two directions of prisms, the pupil was generally smaller with prisms than without prisms. The reason for this effect is unclear. The amount of the prism effect in pupil size ranged between - 0.05 to - 0.16 mm in the 6 conditions and were generally significant (p < 0.001), except for the Cross test and base-in prisms (p = 0.078). The average prism effect in pupil size was - 0.1 mm and this could have artifactually produced an eso shift of 3.0 min arc in the recordings of objective fixation disparity, based on data in the literature (see Methods [93, 94]). When divided by the pooled group standard deviation SD_pooled_ of objective fixation disparity (which was in the range of 15 to 27 min arc), the estimated prism effect size resulting from a potential pupillary artifact could be 0.11 to 0.20, which is smaller than the observed prism effects sizes in objective fixation disparity. Such small pupil-induced effect sizes may be included as random error variance in the correlations of individual prism effects sizes (Fig. 9 and Fig. 10). But this did not prevent the significant correlations due to the much larger range of about 4 for base-in prisms and about 2 for base-out prisms (Fig. 10). Further, the subjective fixation disparity is measured with the psychophysical nonius procedure and is therefore unaffected by the pupil size. Therefore, the significant correlations between the two types of fixation disparity (Fig. 7, Fig. 8 and Fig. 10) are robust with respect to a potential pupillary artifact. These correlations of individual prism effects represent the main result in the base-in group.

The base-out group had mean pupil effects due to wearing prisms of - 0.092, - 0.093, and ‑ 0.098 mm (in the three types of tests), which could produce an artifactual eso shift in objective fixation disparity of 2.9 min arc while exo shifts by - 19.79, - 10.41, and - 3.74 min arc were found. Thus, these latter shifts may – if any – be underestimations since the potential pupillary artifacts may produce an eso shift.
